# Incident sarcopenia in hospitalized older people: A systematic review

**DOI:** 10.1371/journal.pone.0289379

**Published:** 2023-08-02

**Authors:** Safiyyah Nurnajah Wan, Chiann Ni Thiam, Qi Xuan Ang, Julia Engkasan, Terence Ong

**Affiliations:** 1 Department of Medicine, Faculty of Medicine, Universiti Malaya, Wilayah Persekutuan Kuala Lumpur, Malaysia; 2 Department of Medicine, Hospital Sultanah Bahiyah, Alor Setar, Kedah Darul Aman, Malaysia; 3 Department of Rehabilitation Medicine, Universiti Malaya, Wilayah Persekutuan Kuala Lumpur, Malaysia; University of Liege: Universite de Liege, BELGIUM

## Abstract

Hospitalization has been associated with the development of sarcopenia. This study aimed to examine the new incidences of hospital sarcopenia, associated risk factors and health outcomes, as defined by internationally recognized diagnostic criteria in hospitalized older people. Pre-defined search terms were run through five databases. Six studies that assessed sarcopenia on two separate time points during hospitalization on older inpatients were included. Prevalence of sarcopenia varied from 14.1% to 55% depending on diagnostic criteria and cut-off points used. New sarcopenia occurred between 12% to 38.7% patients following hospitalization. Risk factors were older age, longer duration of bed rest, lower baseline body mass index, cognitive impairment and activities of daily living disability. None of the studies reported health outcomes associated with newly developed sarcopenia in hospital.

## Introduction

As humans age, muscle strength reduces at a rate of 3% per year after the 6^th^ decade [1]. Sarcopenia occurs when skeletal muscle mass, strength and physical performance decline at a faster rate [2]. Within the last two decades, sarcopenia discourse had moved towards the distinction between chronic sarcopenia that is often associated with older age; with acute sarcopenia [2, 3]. Due to the distinction between the two as well as specific challenges pertaining to the latter phenomenon, studies have encouraged adopting a different approach to sarcopenia in acute settings [4]. Among older hospital patients screened with the SARC-F questionnaire on admission, 64.5% of them were considered at risk for sarcopenia [5]. In a separate study that categorized hospitalized patients based on ambulatory status, sarcopenia was prevalent in 57.9% patients who could walk independently, 76.1% who walk with aids, 89.4% in wheelchair users, and 91.7% in immobile groups [6]. Sarcopenia is associated with poor outcomes including increased risk of mortality, falls, incidence of hospital admissions, and disability [7].

However, many studies investigating sarcopenia in hospital among older adults only reported its prevalence at a single time point during their inpatient stay [8–13]. Not much has been reported about sarcopenia that newly develops or worsens in hospital. Muscle assessments if they were repeated only reported changes in individual muscle parameters instead of the whole diagnosis of sarcopenia [14–16]. Associations were also made between risk factors and outcomes with individual muscle parameters as a surrogate marker for sarcopenia diagnosis [17] or sarcopenia diagnosed at a single time point during admission [8, 18]. This systematic review aimed to analyze current literature that described newly developed sarcopenia among older adults admitted to hospital, risk factors for these changes and its association with healthcare outcomes.

## Methods

Five electronic databases including MEDLINE, Cochrane Library, Cumulative Index to Nursing and Allied Health Literature (CINAHL), MyMedR, and Web of Science were searched for eligible studies which involved general unplanned non-critical care hospitalized adult population with mean age of more than 50 years. The diagnosis of sarcopenia was based on internationally recognized criteria; European Working Group for Sarcopenia in Older People (EWGSOP) [2], EWGSOP2 which was updated in 2019 [19], the Foundation for National Institutes of Health Sarcopenia Project [20], International Working Group for Sarcopenia [21], and Asian Working Group for Sarcopenia (AWGS) [22] updated in 2019 [23] (detailed in S1 Appendix). Only studies that repeated the same sarcopenia assessments on two separate occasions during hospitalization were included. Observational and interventional studies were included if there was a usual or standard care arm (control group). Studies were excluded if either change in a single muscle parameter was assessed, or muscle assessments were not aligned with a required sarcopenia diagnosis. Studies that were not in English, have a patient cohort that did not reflect the general hospital population (such as those in intensive care unit, patients with specific diagnosis, and those with specific post-operative procedures) were also excluded. Grey literature and trial registries were not searched.

The search strategy involved keywords related to sarcopenia and hospital. Study intervention keywords include: hospital, inpatient, acute care, admission. Study outcome keywords included: variable US/UK spelling of sarcop*nia. The decision to restrict the search strategy to ‘sarcopenia’ alone was in view of the recognition of ‘sarcopenia’ being listed in ICD-10-CM diagnosis code (M62.84) [24]. The search ran from 2010 onwards (as the earliest diagnostic consensus was published in 2010) until May 15^th^ 2023. Citation searching from primary papers for eligible studies was also performed. Examples of the search strategy used for the databases are included in S2 Appendix.

The studies were also appraised independently by WSN, TCN and AXQ for quality assessment. Assessment tools from the Joanna Briggs Institute (JBI) were used to appraise the quality of the included papers [25]. The JBI forms used for different study designs (cohort studies and clinical trial studies) are included in S5 Appendix. JBI does not stratify study quality into poor, moderate and good [26]. Should reviewers decide to proceed with this, it does recommend reviewers to then decide on the thresholds depending on the context of their review. Several studies that utilized JBI grading for their reviews were used as guidance to determine these thresholds [27–30]. There was a lack of consistency in how the JBI was used to categorize study quality. Hence, this study’s researchers met prior to the start of the search, and through an iterative process determined the grading thresholds as follows: as most studies [27, 28, 30] agreed that less than 50% completion of domains were considered to have high risk of bias, completion of 1–5 domains in this review was graded as poor, 6–8 domains was graded as moderate, 9–11 domains was graded as good for cohort studies. As for clinical trial studies, completion of 1–6 domains (less than 50%) was considered poor, 7–11 domains was considered moderate, and 12–13 domains was considered good.

Synthesis of the overall data began with a description of the overall findings and then individual findings of the studies included. Analysis was done based on predetermined subheadings of incidence of new sarcopenia, risk factors and outcomes. Synthesis considered different diagnostic criteria for sarcopenia (type of muscle assessment, equipment used for each assessment, calculation and cut-off point) used in each study. Any categorical or continuous data, or outcomes measured from similar studies using comparable diagnostic criteria would be pooled together. Outcomes of interest that were not identified in the review were also reported.

The protocol for this review was registered with PROSPERO (Registration No. CRD42021234792). No major changes had since been made to the protocol.

## Results

### Search strategy

**Fig 1:**
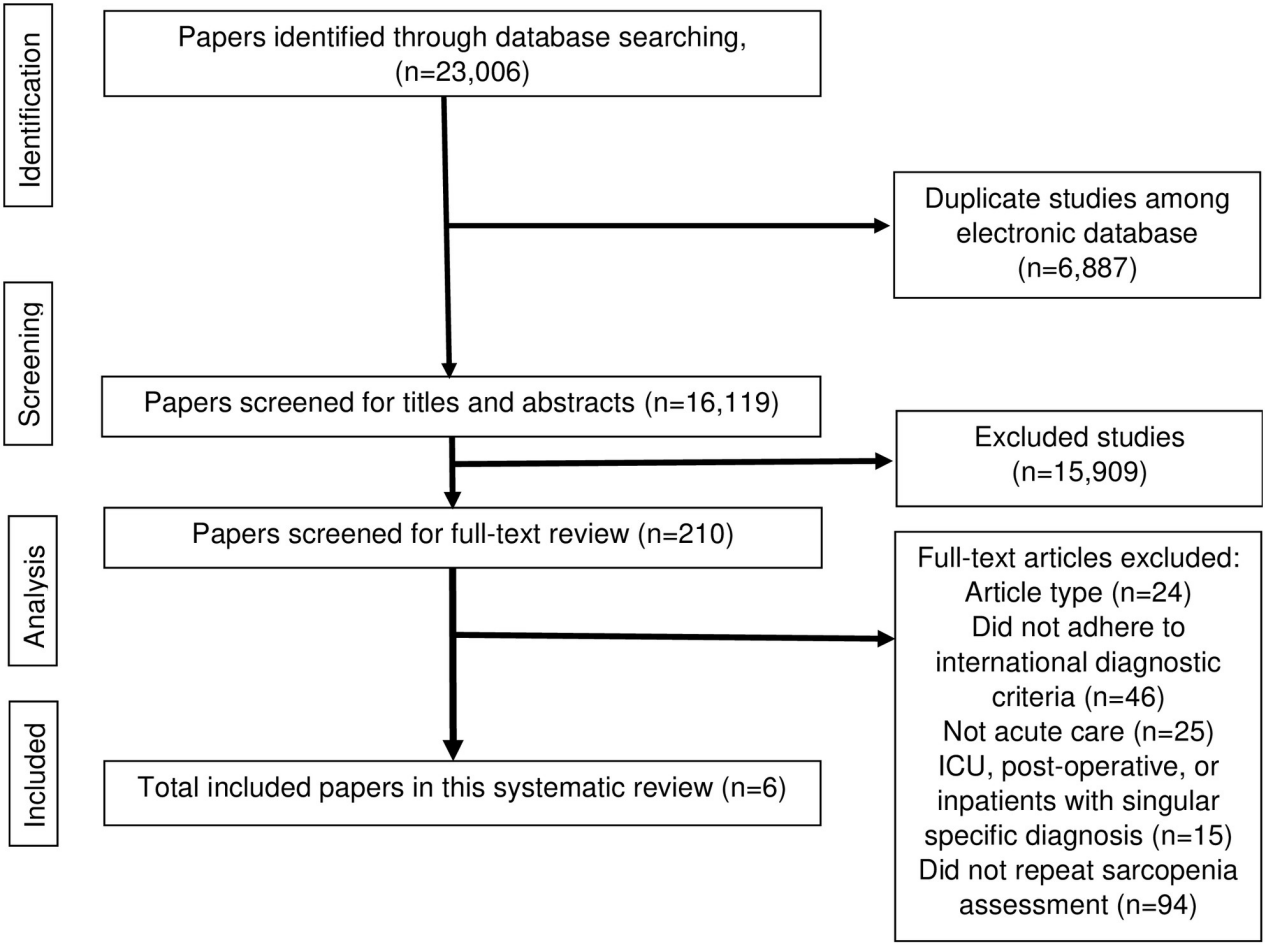
PRISMA diagram for the systematic review.

From 23,006 studies, six studies [31–36] were included. The flow diagram for the search strategy is shown in Fig 1.

### Included studies

Five of the studies were cohort studies [31–33, 35, 36] and one was a double blinded randomized clinical trial [34]. The papers included a total of 1486 participants from 24 hospitals in six countries, 43.5% of which were female. The mean age for all the participants were 78.1 years. The studies involved participants recruited from geriatric medicine wards [31, 35], general medical wards [32, 34–36], surgical wards [36] and cardiology wards [31, 33] Mean duration of stay for the study participants were 14 days. Details of demographic particulars of included studies are presented in Table 1.

**Table 1:** Studies included in the systematic review.

Author, year	Country	Clinical setting	Sarcopenia diagnosis	Muscle mass assessment	Muscle strength assessment	Physical performance assessment	Sample size, gender	Age	Time to follow up and duration of stay
**Aarden et al., 2021** [31]	Netherlands	Geriatric medicine and cardiology wards from 6 hospitals	EWGSOP2	BIA using Quadscan 4000 (supine position), SMM derived from Janssen calculation, SMI adjusted for height (cut-off ≤10.7 kg/m^2^ for men and ≤6.75kg/m^2^ for women)	HGS using Jamar dynamometer, (cut-off <27kg for men and <16kg for women)	SPPB (cut-off ≤8 points); which included Gait speed (cut-off <1.2m/s) Chair stand (cut-off >13.7 seconds)	343, 49.3% female	Mean age: 79.3 SD: ± 6.6 years	Participants assessed within 48 hours of admission and on discharge
									Median duration of hospital stay: 5.7 days (IQR = 4.8)
**Ballesteros-pomar et al., 2021** [32]	Spain	Medical wards from a single hospital	EWGSOP2	BIA by BIA 101^®^ Akern Bioresearch SRL, Pontassieve, Florence, Italy (position not specified), ALMI taken using Scafoglieri et al. calculation, ALMI adjusted for height (cut-off <7kg/m^2^ for men and <5.5kg/m^2^ for women)	HGS using DynEx Hand Grip electronic dynamometer (cut-off <27 kg for men and <16kg for women)	30-second chair stand test (cut-off based on age-dependent CDC guideline for STEADI initiative)	200, 50% female	Median age (IQR) for male: 76.9 (15.0) years, median age for female: 73.9 (30.0) years	Participants assessed within 48 hours of admission, after 1 week of admission, and upon discharge
									Median duration of hospital stay: 7.0 days (IQR = 7.0)
**Gade et al., 2019** [34]	Denmark	Medical ward from 3 hospitals	EWGSOP2	BIA by tetra polar 8-Point Tactile Electrode System (InBody-230) (standing upright), LBM taken (cut-off points not specified)	HGS using DHD-1 digital hand dynamometer (cut-off points not specified)	Gait speed, (cut-off points not specified)	148, 67.6% female	Mean age: 84.2 years SD: ± 6.3 years	Participants assessed within 72 hours of admission, repeated 72 hours prior to discharge
									Median duration of hospital stay: 6 days (IQR = 14)
**Harada et al., 2017** [33]	Japan	Cardiology ward from a single hospital	AWGS	BIA by InBody S10 (Biospace, Tokyo, Japan) (position not specified). Appendicular skeletal muscle mass index (ASMMI) adjusted for height (cut-off <7kg/m^2^ for men and <5.7kg/m^2^ for women)	HGS using Smedley hand dynamometer (cut-off <26 kg for men and <18kg for women)	Gait speed (cut-off <0.8m/s)	322, 42% female	Mean age: 72 SD: ± 12 years	Participants assessed on admission (time not specified) and on discharge (time not specified)
									Mean duration of hospital stay: 32 SD: ±23 days
									Median duration of hospital stay: 7.0 days (IQR = 7.0)
**Martone et al., 2017** [35] (part of GLISTEN study)	Italy	Geriatric and medical wards from 12 hospitals	EWGSOP	BIA by Quantum/S Bioelectrical Body Composition Analyser (supine position), SMM derived using Janssen calculation, SMI adjusted for height (cut-off ≤8.87kg/m^2^ in men and ≤6.42kg/m^2^ in women)	HGS using JAMAR dynamometer (cut-off points from InCHIANTI study, otherwise not specified)	Gait speed over 4m course (cut-off <0.8m/s)	394, 53.6% female	Mean age: 81.0 SD: ± 6.8	Participants assessed within 48 hours of admission and repeated upon discharge (time not specified)
									Mean duration of hospital stay: 10.2 SD: ±8.1 days
**Welch et al., 2022** [36]	United Kingdom	Emergency surgery and medical inpatients recruited from surgical and medical wards	EWGSOP2	BIA by Bodystat Quadscan 4000 (supine position), SMM derived using Sergi equation (cut off <20 kg in men, <15 kg in women), and/or anterior thigh thickness (BATT) (<5.44 cm in men, <3.85 cm in women)	HGS using Jamar dynamometer, (cut-off <27kg for men and <16kg for women)	SPPB and gait speed over 4m, (cut off not specified)	79, 39.2% female	Mean age: 79.1 SD:± 6.6	Participants assessed within 48 h of surgery in the emergency surgery cohort, and within 48 h of admission in the medical cohort, then repeated at 7 ± 2 days post-hospitalization/surgery
									Duration of stay for all participants not reported

EWGSOP2—European Working Group on Sarcopenia in Older People 2, EWGSOP—European Working Group on Sarcopenia in Older People, AWGS—Asian Working Group on Sarcopenia, SD—standard deviation, IQR—interquartile range, BIA—Bioelectric impedance analysis, SMM—Skeletal Muscle Mass, SMI—Skeletal Muscle Index, LBM—Lean Body Mass, ASMM—Appendicular Skeletal Muscle Mass, ASMI—Appendicular Skeletal Muscle Index, ALM—Appendicular Lean Mass, ALMI—Appendicular Lean Mass Index, HGS—hand grip strength, CDC—The Centres for Disease Control and Prevention, STEADI—Stopping Elderly Accidents, Deaths and Injuries Initiative, GLISTEN—Gruppo Lavoro Italiano Sarcopenia-Trattamento e Nutrizione, InCHIANTI—"Invecchiare in Chianti" study, SPPB -Short Physical Performance Battery.

### Study quality

Out of 11 components or domains assessed, one study only completed three (3/11) domains [33] from the Joanna Briggs Institute Checklist for Analytical Cohort Studies, one study completed six (6/11) domains [32], one study completed seven (7/11) domains [35], and two studies completed eight (8/11) domains. One clinical trial study [37] only completed eight out of 13 domains of the Checklist for Randomized Controlled Trials. The full table is included in S5 Appendix. Generally, the included studies were of moderate [31, 32, 34, 35] to poor quality [33]. One of the moderately scored studies combined results from emergency and elective inpatient cohort [36].

### Diagnostic criteria

One study used the AWGS criteria for sarcopenia diagnosis [33], one study used the EWGSOP criteria [35] whereas the other 4 studies used EWGSOP2 criteria [31, 32, 34, 36]. For the measurements of muscle mass, muscle strength and physical performance; all studies used varying equipment, participant positions, calculation methods and cut-off points.

### Prevalence of sarcopenia in hospital

All six articles included in this review reported the prevalence of sarcopenia on admission among participants aged more than 50 years. The prevalence; which were 14.1% [31], 28% [33], 33% [32], 36.3% [35], 50.7% [34] and 55% [36]; were different according to the diagnostic criteria and cut-off points used by each study (Table 1). Only Welch et al., Martone et al., and Ballesteros-Pomar et al. reported on newly developed sarcopenia in hospital which were reported to be 12% [36], 14.7% [35], and 38.7% [32] respectively after 7 days of hospitalization or upon discharge. All three studies employed criteria based on different cut-off reference points. Of these, Martone et al. alone reported on the prevalence of sarcopenia according to gender (men, 15.8% vs women, 13.7%, p = 0.32) [35].

### Sarcopenia during hospitalization

Due to the varied ways which muscle mass were assessed; different outcomes were reported. Ballesteros-Pomar et al. reported a decrease of 118g of appendicular lean mass (ALM) in all participants which was statistically significant (*p* = 0.034) [32], whereas Gade et al. reported a general reduction of lean body mass (LBM) in all participants [34] which was not statistically significant. Martone et al. reported that in more than half of participants that developed new sarcopenia in hospital, skeletal muscle mass (SMM) loss was more than 10% [35]. However, statistical significance was not reported in the study. Welch et al., reported decreasing SMM after one week of hospitalization, but it was not significant [36]. Only Aarden et al. compared muscle index, and found that there was no change in mean of skeletal muscle mass index (SMI) between admission and discharge in all participants [31]. Findings are summarised in the table below (Table 2).

**Table 2:** Muscle changes during hospitalisation reported in included studies.

Author, year	Prevalence	Muscle mass change	Muscle strength change	Physical performance change	New sarcopenia
**Aarden et al., 2021** [31]	14.1%	Mean SMI on admission (kg/m^2^): 7.9, 95% CI: 7.8–8.2 Mean SMI on discharge (kg/m^2^): 7.9, 95% CI: 7.7–8.1	Mean HGS on admission for all patients (kg): 27.1, 95% CI: 26.1–28.1 Mean HGS on discharge for all patients (kg): 27.2kg, 95% CI: 26.1–28.2	Mean chair stand test points on admission for all patients: 1.1, 95% CI: 1.0–1.3 Mean chair stand test points on discharge for all patients: 1.5, 95% CI: 1.3–1.6 Mean gait speed test points on admission for all patients: 1.9, 95% CI: 1.8–2.1 Mean gait speed test points on discharge for all patients: 2.3, 95% CI: 2.2–2.5	Not reported
**Ballesteros-Pomar et al., 2021** [32]	33%	Median ALM change on discharge from admission for all patients (kg):—0.118 (IQR: 0.490) *p* = 0.039	Median HGS change on discharge from admission for all patients (kg):—0.65 (IQR: 4.9) *p* = 0.364	Chair stand test: 75% of all patients abnormal on admission, 81.3% abnormal after 1 week and 82.6% abnormal on discharge Statistical significance was not determined	38.7% from previously non-sarcopenic patients on admission
**Gade et al., 2019** [34]	50.7%	Median LBM change on discharge from admission for all patients (kg):—0.1 (IQR: 2.1) *p* = 0.332	Median HGS change on discharge from admission for all patients (kg):—0.2 (IQR: 4.5) *p* = 0.681	58.7% of all patients had an improvement in chair-stand test compared to admission *p* = 0·348	Not reported
**Harada et al., 2017** [33]	28.0%	Reduced SMI (from 7.5 ± 1.4 to 7.2 ± 1.3kg/m^2^, p<0.01 in those without sarcopenia; and from 5.3 ± 0.8 to 5.2 ± 0.9 kg/m^2^, p>0.05 in those with sarcopenia), among exercise therapy group however this does not reflect normal acute care population	Increased hand grip strength (from 27.3 ± 11.8 to 28.5 ± 11.3kg, p<0.05 in those without sarcopenia; and from 13.6 ± 6.7 to 15.0 ± 7.8kg, p<0.01 in those with sarcopenia) among exercise therapy group, however this does not reflect normal acute care population	Increased gait speed (from 1.18 ± 0.37 to 1.32 ± 0.41m/s, p<0.001 in those without sarcopenia; and from 0.83 ± 0.29 to 0.92 ± 0.29m/s, p<0.01 in those with sarcopenia) among exercise therapy group, however this does not reflect normal acute care population	Not reported
**Martone et al., 2017** [35]	36.6%	More than 50% participants with new sarcopenia had over 10% SMM loss compared to admission, statistical significance not reported	Not reported	Not reported	14.7% from previously non-sarcopenic patients on admission
**Welch et al., 2022** [36]	55%	Marginal mean SMM on admission for males (kg): 21.6 (95% CI: 19.9–23.2) for females (kg): 16.0 (13.9–18.1) Marginal mean SMM after 7 days for males (kg): 21.0 (95% CI: 19.2–22.8) for females (kg): 15.3 (13.1–17.5)	Marginal mean HGS on admission for males (kg): 24.1 (95% CI: 21.1–27.1) for females (kg): 14.7 (95% CI: 11.6–17.7) Marginal mean HGS after 7 days for males (kg): 23.1 (95% CI: 19.8–26.4) for females (kg): 13.4 (95% CI: 10.4–16.3)	Marginal mean gait speed on admission (m/s): 0.65 (95% CI: 0.58–0.73) Marginal mean gait speed after 7 days (m/s): 0.50 (95% CI: 0.43–0.58) Marginal mean SPPB score on admission: 6.19 (95% CI: 5.24–7.32) Marginal mean SPPB after 7 days: 4.25 (95% CI: 3.01–5.85)	12% from previously non-sarcopenic patients on admission

SMI—Skeletal Muscle Index, CI—Confidence interval, HGS—hand grip strength, ALM—Appendicular Lean Mass, IQR—interquartile range, LBM—Lean Body Mass, SMM—Skeletal Muscle Mass, SPPB—Short Physical Performance Battery

All three reports by Ballesteros-Pomar et al., Gade et al. and Welch et al. [32, 34, 36] demonstrated that hand grip strength of participants reduced on discharge, however not adequate to be statistically significant. Aarden et al. [31] reported slight increase (0.1kg) in mean hand grip strength on discharge, but statistical significance was not reported.

There were conflicting reports on physical performance of older patients. This was due to different assessment methods (Aarden et al. used point-based system [31], Ballesteros-Pomar et al. and Gade et al. used duration of completion [32, 34]), and cut-off points used. Ballesteros-Pomar et al. [32] reported that more patients had abnormal chair stand test following hospitalization compared to during admission (82.6% vs 75% respectively), however statistical significance was not reported. Aarden et al. and Gade et al. [31, 34] reported that chair stand test improved upon discharge. Only Aarden et al. [31] reported on gait speed, all of which improved upon discharge. Welch reported that both gait speed and Short Physical Performance Battery (SPPB) score reduced after 7 days of hospitalization, but the changes were not statistically significant. None of the studies described change in severity of sarcopenia throughout hospital admission.

The findings are summarized in Table 2 below.

### Risk factors associated with sarcopenia in hospitalized older people

Only Martone et al. [35] explored the possible risk factors associated with newly developed sarcopenia in hospital. Age was reported to be higher in those that developed sarcopenia (mean (SD) age of participants with new sarcopenia: 82.0 (7.2) years, vs mean (SD) age of participants without sarcopenia: 79.2 (6.2) years, *p*<0.01). Whilst there was no significant association between sarcopenia and length of hospital stay, those that developed sarcopenia had longer days of bed rest (mean (SD) 5.1 (5.3) days) compared to those without sarcopenia (mean (SD) 3.2 (6.7) days) (*p* = 0.02). Cognitive impairment based on the Short Portable Mental Status Questionnaire (SPMSQ) was associated with a higher probability of developing sarcopenia during hospitalization (odds ratio (OR) 1.03; 95% confidence interval (CI): 0.97–1.32, *p*<0.001). There was decreased risk of developing sarcopenia among patients with higher baseline body mass index (BMI) (mean (SD) 25.0 (3.8) kg/m^2^) compared to those with lower baseline BMI (mean (SD) 27.6 (4.9) kg/m^2^) (OR: 0.92; 95% CI: 0.86–0.98) (*p* = 0.02). Activities of daily living (ADL) dependency was also found to be associated with a higher probability with developing sarcopenia during hospitalization (OR: 1.23; 95% CI: 1.01–1.49, *p* = 0.02). The same study reported no significant association between newly developed sarcopenia with gender, polypharmacy, serum albumin, hemoglobin level or comorbidities (based on Charlson Comorbidity Index). There was no other reported association between newly developed sarcopenia in hospital with malnutrition, frailty, levels of physical activities, or falls.

### Health outcomes associated with sarcopenia

Ballesteros-Pomar et al. [32] reported on health outcomes associated with sarcopenia diagnosis on admission. It was reported that a sarcopenia diagnosis on admission using the EWGSOP2 criteria was associated with a poor quality of life measured by EuroQol 5 Dimension 5 Level (EQ-5D-5L) (EuroQol Visual Analogue Scale median total score 50.0 (IQR: 18.0) in those with sarcopenia vs 60.0 (IQR: 30.0) in those without, *p*<0.001), higher readmission rate in 3 months (OR: 2.25; 95% CI: 1.52–2.99, *p* = 0.03) and mortality (inpatient and outpatient) over 3 months (OR: 8.16; 95% CI: 6.52–9.79, *p* = 0.012). None of the studies reported on mortality, inpatient complications, readmission rate, change in functional ability on discharge and quality of life of participants with newly developed sarcopenia during hospitalization.

## Discussion

This systematic review analyzed available literature surrounding sarcopenia that developed during hospitalization. There is paucity of literature on the topic when the diagnosis of sarcopenia was used. An extensive search was done involving six different databases which identified only 5 studies with poor to moderate quality. This review found the prevalence of sarcopenia on admission to be between 14.1% to 55% based on the recognized international criteria; within range of reported sarcopenia prevalence in hospital based on EWGSOP1 criteria which are 33.8% in Italy [8], and 34.1% in Norway [9]; as well as EWGSOP2 which are 50.6% [10] and 25.5% [11] both in Italy. In Asia, the prevalence of sarcopenia among older patients admitted to cardiology wards based on AWGS were 38.6% [37] in Japan and 34.3% [12] in Vietnam; compared to 28.0% in the included study [33]. It should be noted that the setting used in the studies included in this review also varied; such as emergency department, surgical, medical cardiology and geriatric departments; which could contribute to the wide range of prevalence. However, this also indicated that this condition did occur across many disciplines. Apart from this, instruments used to assess sarcopenia also varied from study to study. The assessments were also made and repeated at different time points during hospitalization which can cause the incidence to vary. This also highlights the possibility that the instruments used were not sensitive enough to assess changes over a short duration of time. Importantly, this review found that there was lack of harmonization in the utilization of sarcopenia diagnostic criteria, devices used for individual muscle assessment, as well as cut-off points; which made it difficult to compare and reconcile the differences in prevalence. This challenge in standardizing the diagnostic criteria for sarcopenia remains pervasive as reported in other existing literature [38].

None of individual muscle changes were statistically significant throughout hospitalization in this review. In studies that examined change in muscle mass, the change reported during hospitalization in patients admitted to hospital were found to be not statistically significant [39, 40]. Even so, there are limitations in using body impedance analysis particularly among older people in acute care setting [41] as its accuracy can be affected by other factors such as volume status. For muscle strength, most studies reported no significant change of hand grip strength during hospitalization [15, 16]. On the other hand, physical performance assessments in existing studies demonstrated that participants had significantly improved Timed Up and Go [15], Short Physical Performance Battery test [42], and 30-second chair stand test [43]. However, all these studies varied greatly in clinical settings, assessment methods and cut-off points. This review demonstrated that when comprehensive reassessment was done to make a specific diagnosis of sarcopenia, articles reported that 12% [36] to 38.7% [32] of previously non-sarcopenic patients subsequently developed sarcopenia. This suggests that depending on single muscle component as a clinical indicator alone is not reliable and may contribute to inaccurate diagnosis of sarcopenia during hospitalization. Rather, the assessment and re-assessment of sarcopenia should encompass all three muscle components particularly throughout inpatient hospital management.

This review found that those who developed sarcopenia following hospitalization were significantly older. This corresponds with existing literature which described increased prevalence of sarcopenia with increased age [44]. Although two articles included in this review reported that there was no significant association between newly developed sarcopenia with length of hospital stay, Martone et al. reported that there was significant association with days of bed rest [35] for an average of 5 days, compared to 3 days in those that did not develop acute sarcopenia. This is in line with current evidence that showed immobilization for 5 days in healthy older people resulted in reduced muscle protein synthesis, manifesting as decreased lean mass on dual-energy x-ray absorptiometry (DXA) [45]. Similar to other studies [46, 47], this review found a negative relationship between baseline body mass index with sarcopenia. Cognitive impairment and ADL disability were also risk factors for developing sarcopenia [35], as supported by other studies in acute care setting [47–50].

There is huge absence in examining risk factors and outcomes associated with developing sarcopenia or worsening of sarcopenia in hospital in existing literature. Few studies explored the association of new sarcopenia or worsening sarcopenia with nutrition status, falls, frailty, levels of physical activities, mortality, rate of readmission, development of inpatient complications, change in functional abilities upon discharge, or quality of life. Rather, other studies have reported poor clinical outcomes associated with low muscle mass or low grip strength alone [51]. Similar long term health outcomes should also be anticipated in patients that developed sarcopenia during hospitalization.

One of the strengths of this review was that it examined sarcopenia as a diagnosis and not individual muscle parameters. This is clinically important as the ultimate aim is to manage a disease rather than a component of it. This review was comprehensive as it identified as many bibliographic databases as possible. This review also employed wide inclusion criteria.

However, a systematic review of sarcopenia prevalence on admission, during hospitalization, or at discharge alone was not done thus likely excluding several studies. This review was not able to comprehensively conclude an overall sarcopenia prevalence in hospital. Hence, it is difficult determine if the prevalence of sarcopenia identified at all these different time points were associated with specific outcomes or particular risk factors. Similarly, there would be a lot more literature on individual muscle parameter changes [14–16], which suggests that sarcopenia during acute hospitalization does occur. Currently there is insufficient evidence to indicate the exact point at which the muscle parameters fall below threshold during hospitalization to warrant a diagnosis of sarcopenia. This review also only included studies that were published in English and therefore could have missed eligible studies published in other languages. Researchers for this review were English speakers only and lacked funds for translation services. Reports had previously demonstrated that the exclusion of studies in language other than English did not impact on the conclusion of systematic reviews [52, 53]. Only published work were included in this review. This study focused on the sarcopenia phenomenon in hospital and not on any specific healthcare intervention. Hence, gathering all reported participant characteristics that was also deemed more reliable was more likely in peer-reviewed published work that what would be reported in either grey literature or trial registries. Additionally, trial registries, if included, were found to have little impact on qualitative interpretation of study results [54].

It should be highlighted that the only study found in this review that studied incidence and risk factors of sarcopenia in hospital involved a homogenously Caucasian sample [35]. Muscle has geographical variation, and the recognized consensus AWGS alone differs from EWGSOP2 criteria in terms of cut-off points. For instance, hand-grip strength among Malaysians have been shown to be up to 1.5 times smaller than that of Western population [55]. This review was not able to explore this further due to insufficient evidence.

This review highlighted the paucity of literature in this field. More research should be done based on a standardized criteria and cut-off points to identify the incidence of acute sarcopenia in older people admitted to hospital, risk factors and clinical outcomes. Analysis on different gradations of muscle changes particularly with respect to severity of sarcopenia in hospital is also lacking. A minimum core dataset for future researchers embarking on hospitalization sarcopenia investigation should be defined and researchers should be obligated to fulfil a minimum set of data with standardized assessment methods. This is to ensure that better data is obtained from which standardized and objective comparisons can be made. In addition, future studies should be done on different ethnicities and geographical locations to reflect different variations of muscle pathology.

## Conclusion

Sarcopenia develops in older people following hospitalization. This review demonstrated the general lack of harmonization on diagnostic criteria and cut-off points used for the assessment of sarcopenia during hospitalization in older people in current literature. There are also very few studies that explored associations between factors contributing to newly developed sarcopenia in hospital and subsequent health outcomes. Research to address this gap is required to better understand this phenomenon to improve its management and deliver better healthcare outcomes.

## Supporting information

S1 Appendix Sarcopenia definitions. (DOCX)

S2 Appendix Search strategy. (DOCX)

S3 Appendix References for full texts screened. (DOCX)

S4 Appendix Example of extracted data table. (DOCX)

S5 Appendix Quality assessment table and JBI appraisal forms. (DOCX)

S6 Appendix AMSTAR 2 checklist. (DOCX)

S1 Data (XLSX)
