# Supplementary material for: Incident sarcopenia in hospitalized older people: A systematic review
Source: PLoS One. 2023 Aug 2;18(8):e0289379. doi: 10.1371/journal.pone.0289379 (PMC10395895; doi:10.1371/journal.pone.0289379)
Supplement: S1 Appendix — (DOCX) [file pone.0289379.s001.docx]

**Appendix 1. Sarcopenia definitions**

Table 3: Comparison between cut off points and diagnostic criteria from IWGS, EWGSOP 1 and 2, AWGS 2014 and updated 2019, and FNIH, with added row for Sarcopenia

Definition and Outcomes Consortium (SDOC) consensus.

| Author/ Association/ Year | Definition | Muscle strength | Muscle quality/quantity | Physical performance |
| --- | --- | --- | --- | --- |
| Fielding et al, 2011  IWGS | Low whole body or appendicular fat-free mass  AND  Poor physical functioning | NA | Appendicular fat lean mass/height² (ALM/ height²) of ≤7.23 kg/m² and for men and ≤5.67 kg/m² for women | Gait speed of less than 1 m/s |
| Cruz-Jentolf et al, 2010  EWGSOP1 | Low muscle mass  AND, either;  Low muscle strength  OR  Low physical performance | Cut-off points not specified, but generally at two standard deviations below the mean reference value (normative data) | Cut-off points not specified, but generally at two standard deviations below the mean reference value (normative data) | Cut-off points not specified, but generally at two standard deviations below the mean reference value (normative data) |
| Cruz-Jentolf et al, 2018  EWGSOP2 | Low muscle mass alone is probable sarcopenia  Confirmed sarcopenia:  Low muscle mass  AND, either;  Low muscle strength  OR  Low physical performance  Severe sarcopenia is when all three components are present | Handgrip strength of <27 kg for men and <16 kg for women  OR  5-times Chair stand test ≥15s | Bioelectrical impedance analysis: appendicular skeletal muscle mass of <20 kg for men and <15 kg for women  Appendicular skeletal muscle mass/height² with cut-off points for men <7.0 kg/m² and <5.5 kg/m² for women | Gait speed ≤0.8 m/s, short physical performance battery (SPBB) ≤8 points, timed up and go test (TUG) ≥20s |
| Chen et al, 2014 AWGS | Low muscle mass  plus  low muscle strength and/or low physical performance | <26 kg for men and <18 kg for women | 7.0 kg/m2 for men and 5.4 kg/m2 for women by using dual X-ray absorptiometry, and 7.0 kg/m2 for men and 5.7 kg/m2 for women by using bioimpedance analysis | <0.8 m/s |
| Chen et al, 2019  AWGS (updated) | Possible sarcopenia: Handgrip strength  (M: <28 kg, F: <18 kg) OR  5-time chair stand test (≥12 s)  Sarcopenia:  Low muscle mass  AND, either;  Low muscle strength  OR  Low physical performance  Severe sarcopenia is when all muscle parameters are low | Handgrip strength <28 kg for men and <18 kg for women | Bioelectrical impedance analysis <7.0 kg/m² for men and <5.7 kg/m² for women | 6-m walk of <1.0 m/s  or 5-time chair stand test: ≥12 s  or Short Physical Performance Battery: ≤9 |
| Studenski et al, 2014  (FNIH) | FNIH avoided the use of the term ‘sarcopenia’; instead;  handgrip strength < 26 kg and Appendicular Lean Mass adjusted for Body Mass index < 0.789 to define “weakness and low lean mass”; combined with gait speed ≤0.8 m/s the condition is called “slowness with weakness and low lean mass” | Handgrip strength <26 kg | Appendicular lean mass adjusted for body mass index < 0.789 | Gait speed ≤0.8 m/s |
| Bhasin et al., 2020 (SDOC consensus) | Both weakness defined by low grip strength and slowness  defined by low usual gait speed should be included in the  definition of sarcopenia | Not specified | Not specified, but consensus agreed that lean mass by DXA is not a good predictor of falls, hip fracture and mortality | Not specified |
