# Supplementary material for: Incident sarcopenia in hospitalized older people: A systematic review
Source: PLoS One. 2023 Aug 2;18(8):e0289379. doi: 10.1371/journal.pone.0289379 (PMC10395895; doi:10.1371/journal.pone.0289379)
Supplement: S2 Appendix — (DOCX) [file pone.0289379.s002.docx]

**Appendix 2. Search strategy**

CINAHL

(Sarcopenia OR sarcopaenia) AND (hospital OR admission OR acute care OR inpatient)

Filters applied to exclude studies:

- Published before 2010
- Not English
- Not academic journals
- Age less than 45

Web of Science

(Sarcopenia OR sarcopaenia) AND (hospital OR admission OR acute care OR inpatient)

Filters applied to exclude studies:

- Published before 2010
- Article types not academic journals
- Not English
- Conference titles
- Book chapters

MEDLINE

(Sarcopenia OR sarcopaenia) AND (hospital OR admission OR acute care OR inpatient)

Filters applied to exclude studies:

- Published before 2010
- Not academic journals
- Not English
- Age less than 45

MyMedR

(Sarcopenia OR sarcopaenia) AND (hospital OR admission OR acute care OR inpatient)

COCHRANE

(Sarcopenia OR sarcopaenia) AND (hospital OR admission OR acute care OR inpatient)

Filters applied to exclude studies:

- Published before 2010
