# Supplementary material for: Incident sarcopenia in hospitalized older people: A systematic review
Source: PLoS One. 2023 Aug 2;18(8):e0289379. doi: 10.1371/journal.pone.0289379 (PMC10395895; doi:10.1371/journal.pone.0289379)
Supplement: S3 Appendix — (DOCX) [file pone.0289379.s003.docx]

**Appendix 3. References for full texts screened**

1. Abay RJY, Gold LS, Cawthon PM, Andrews JS. Lean mass, grip strength, and hospital-associated disability among older adults in Health ABC. Alzheimers & Dementia.
2. Abbas H, Perna S, Shah A, Al-Mannai M, Gasparri C, Infantino V, et al. Risk factors for 5-year mortality in a cohort of elderly patients with sarcopenia. Exp Gerontol. 2020;136:110944.
3. Agosta L, Bo M, Bianchi L, Abete P, Belelli G, Cherubini A, et al. Polypharmacy and sarcopenia in hospitalized older patients: results of the GLISTEN study. Aging clinical and experimental research. 2019;31(4):557-9.
4. Akan B. Influence of sarcopenia focused on critically ill patients. Acute and critical care. 2021.
5. Aliberti MJR, Szlejf C, Covinsky KE, Lee SJ, Jacob-Filho W, Suemoto CK. Prognostic value of a rapid sarcopenia measure in acutely ill older adults. Clinical nutrition (Edinburgh, Scotland). 2020;39(7):2114-20.
6. Anani S, Goldhaber G, Brom A, Lasman N, Turpashvili N, Shenhav-Saltzman G, et al. Frailty and Sarcopenia Assessment upon HospitalAdmission to Internal Medicine Predicts Length ofHospital Stay and Re-Admission: A ProspectiveStudy of 980 Patients. Journal of clinical medicine. 2020;9(8).
7. Antunes AC, Araujo DA, Verissimo MT, Amaral TF. Sarcopenia and hospitalisation costs in older adults: a cross-sectional study. Nutrition & Dietetics. 2017;74(1):46-50.
8. Asaoka D, Takeda T, Inami Y, Abe D, Shimada Y, Matsumoto K, et al. Association between the severity of constipation and sarcopenia in elderly adults: A single-center university hospital-based, cross-sectional study. Biomedical Reports. 2021;14(1).
9. Atmis V, Yalcin A, Silay K, Ulutas S, Bahsi R, Turgut T, et al. The relationship between all-cause mortality sarcopenia and sarcopenic obesity among hospitalized older people. Aging clinical and experimental research. 2019;31(11):1563-72.
10. Attaway A, Bellar A, Dieye F, Wajda D, Welch N, Dasarathy S. Clinical impact of compound sarcopenia in hospitalized older adult patients with heart failure. Journal of the American Geriatrics Society. 2021;69(7):1815-25.
11. Aubertin-Leheudre M, Martel D, Narici M, Bonnefoy M. The usefulness of muscle architecture assessed with ultrasound to identify hospitalized older adults with physical decline. Experimental gerontology. 2019;125:110678.
12. Avrutin E, Moisey LL, Zhang R, Khattab J, Todd E, Premji T, et al. Clinically Practical Approach for Screening of Low Muscularity Using Electronic Linear Measures on Computed Tomography Images in Critically Ill Patients. JPEN Journal of Parenteral & Enteral Nutrition. 2018;42(5):885-91.
13. Baggerman MR, van Dijk DPJ, Winkens B, van Gassel RJJ, Bol ME, Schnabel RM, et al. Muscle wasting associated co-morbidities, rather than sarcopenia are risk factors for hospital mortality in critical illness. Journal of critical care. 2020;56:31-6.
14. Bai H-J, Sun J-Q, Chen M, Xu D-F, Xie H, Yu Z-W, et al. Age-related decline in skeletal muscle mass and function among elderly men and women in Shanghai, China: a cross sectional study. Asia Pacific journal of clinical nutrition. 2016;25(2):326-32.
15. Barreto EF, Kanderi T, DiCecco SR, Lopez-Ruiz A, Poyant JO, Mara KC, et al. Sarcopenia Index Is a Simple Objective Screening Tool for Malnutrition in the Critically Ill. JPEN J Parenter Enteral Nutr. 2019;43(6):780-8.
16. Baylis D, Syddall HE, Jameson KA, Cooper C, Lord JM, Roberts HC, et al. Cachexia, sarcopenia, inflammaging and outcomes in hospitalised older people (the CaSIO study): Study protocol and preliminary results. European Geriatric Medicine. 2015;6(5):495-501.
17. Bayraktar E, Tasar PT, Binici DN, Karasahin O, Timur O, Sahin S. Relationship between sarcopenia and mortality in elderly inpatients. The Eurasian Journal of Medicine. 2020;52(1):29.
18. Beaudart C, Zaaria M, Pasleau F, Reginster J-Y, Bruyère O. Health Outcomes of Sarcopenia: A Systematic Review and Meta-Analysis. PloS one. 2017;12(1):e0169548.
19. Bellanti F, Lo Buglio A, Quiete S, Pellegrino G, Dobrakowski M, Kasperczyk A, et al. Comparison of three nutritional screening tools with the new glim criteria for malnutrition and association with sarcopenia in hospitalized older patients. Journal of clinical medicine. 2020;9(6):1898.
20. Bellanti F, Buglio AL, Stasio ED, Bello Gd, Tamborra R, Dobrakowski M, et al. An open-label, single-center pilot study to test the effects of an amino acid mixture in older patients admitted to internal medicine wards. Nutrition (Burbank, Los Angeles County, Calif). 2020;69:110588.
21. Benton E, Liteplo AS, Shokoohi H, Loesche MA, Yacoub S, Thatphet P, et al. A pilot study examining the use of ultrasound to measure sarcopenia, frailty and fall in older patients. The American journal of emergency medicine. 2020.
22. Beretta MV, Dantas Filho FF, Freiberg RE, Feldman JV, Nery C, Rodrigues TC. Sarcopenia and Type 2 diabetes mellitus as predictors of 2-year mortality after hospital discharge in a cohort of hospitalized older adults. Diabetes research and clinical practice. 2020;159:107969.
23. Bernabeu-Wittel M, González-Molina Á, Fernández-Ojeda R, Díez-Manglano J, Salgado F, Soto-Martín M, et al. Impact of Sarcopenia and Frailty in a Multicenter Cohort of Polypathological Patients. Journal of clinical medicine. 2019;8(4).
24. Bertschi D, Kiss CM, Beerli N, Kressig RW. Sarcopenia in hospitalized geriatric patients: insights into prevalence and associated parameters using new EWGSOP2 guidelines. European journal of clinical nutrition. 2020.
25. Bian A, Ma Y, Zhou X, Guo Y, Wang W, Zhang Y, et al. Association between sarcopenia and levels of growth hormone and insulin-like growth factor-1 in the elderly. BMC musculoskeletal disorders. 2020;21(1):214.
26. Bianchi L, Abete P, Bellelli G, Bo M, Cherubini A, Corica F, et al. Prevalence and clinical correlates of sarcopenia, identified according to the EWGSOP definition and diagnostic algorithm, in hospitalized older people: the GLISTEN study. Journals of Gerontology Series A: Biomedical Sciences and Medical Sciences. 2017;72(11):1575-81.
27. Bianchi L, Maietti E, Abete P, Bellelli G, Bo M, Cherubini A, et al. Comparing EWGSOP2 and FNIH Sarcopenia Definitions: Agreement and 3-Year Survival Prognostic Value in Older Hospitalized Adults: The GLISTEN Study. Journals of Gerontology Series A: Biological Sciences & Medical Sciences. 2020;75(7):1331-7.
28. Bieger P, Sangali TD, Ribeiro É CT, Schweigert Perry ID, Souza GC. Association of phase angle values and sarcopenia in older patients with heart failure. Nutr Clin Pract. 2023;38(3):672-85.
29. Borges RC, Correa DI, Correa LJS, Colombo AS, Carvalho CRF. Prevalence and Factors Associated with Sarcopenia in Hospitalized Elderly Patients. Aging Medicine and Healthcare. 2022;13(2):51-9.
30. Bosaeus I, Wilcox G, Rothenberg E, Strauss BJ. Skeletal muscle mass in hospitalized elderly patients: comparison of measurements by single-frequency BIA and DXA. Clinical nutrition (Edinburgh, Scotland). 2014;33(3):426-31.
31. Bouillanne O, Curis E, Hamon-Vilcot B, Nicolis I, Chretien P, Schauer N, et al. Impact of protein pulse feeding on lean mass in malnourished and at-risk hospitalized elderly patients: A randomized controlled trial. Clinical Nutrition. 2013;32(2):186-92.
32. Byrnes A, Mudge A, Young A, Banks M, Bauer J. Use of hand grip strength in nutrition risk screening of older patients admitted to general surgical wards. Nutrition & Dietetics. 2018;75(5):520-6.
33. Castillo-Olea C, García-Zapirain Soto B, Carballo Lozano C, Zuñiga C. Automatic Classification of Sarcopenia Level in Older Adults: A Case Study at Tijuana General Hospital. International journal of environmental research and public health. 2019;16(18).
34. Cerri AP, Bellelli G, Mazzone A, Pittella F, Landi F, Zambon A, et al. Sarcopenia and malnutrition in acutely ill hospitalized elderly: Prevalence and outcomes. Clinical Nutrition. 2015;34(4):745-51.
35. Chan D-CD, Tsou H-H, Chang C-B, Yang R-S, Tsauo J-Y, Chen C-Y, et al. Integrated care for geriatric frailty and sarcopenia: a randomized control trial. Journal of cachexia, sarcopenia and muscle. 2017;8(1):78-88.
36. Chang S-F, Lin P-L. Systematic Literature Review and Meta-Analysis of the Association of Sarcopenia With Mortality. Worldviews on evidence-based nursing. 2016;13(2):153-62.
37. Chen J-L, Chen D-M, Luo C, Sun Y, Zhao Y-X, Huang C-Q, et al. Fibrinogen, fibrin degradation products and risk of sarcopenia. Clinical Nutrition. 2021;40(8):4830-7.
38. Choi JY, Kim KI, Choi Y, Ahn SH, Kang E, Oh HK, et al. Comparison of multidimensional frailty score, grip strength, and gait speed in older surgical patients. Journal of Cachexia Sarcopenia and Muscle. 2020;11(2):432-40.
39. Choi J-Y, Rajaguru V, Shin J, Kim K-i. Comprehensive geriatric assessment and multidisciplinary team interventions for hospitalized older adults: A scoping review. Archives of Gerontology & Geriatrics. 2023;104:N.PAG-N.PAG.
40. Coleman S, Horgan F, Cunningham C, Murphy N. Progressive resistance training in a postacute, older, inpatient population: a randomised controlled feasibility trial. Age and ageing. 2018;47.
41. Cox MC, Booth M, Ghita G, Wang ZK, Gardner A, Hawkins RB, et al. The impact of sarcopenia and acute muscle mass loss on long-term outcomes in critically ill patients with intra-abdominal sepsis. Journal of Cachexia Sarcopenia and Muscle. 2021;12(5):1203-13.
42. D'Alia S, Guarasci F, Bartucci L, Caloiero R, Guerrieri ML, Soraci L, et al. Hand Grip Strength May Affect the Association Between Anticholinergic Burden and Mortality Among Older Patients Discharged from Hospital. Drugs & Aging. 2020;37(6):447-55.
43. Davis C, Khattab J, Mourtzakis M, Hough CL. Sarcopenia On Admission To The Icu Is Associated With Critical Illness Neuromyopathy. American Journal of Respiratory and Critical Care Medicine. 2016;193.
44. DeAndrade J, Pedersen M, Garcia L, Nau P. Sarcopenia is a risk factor for complications and an independent predictor of hospital length of stay in trauma patients. The Journal of surgical research. 2018;221:161-6.
45. Deer RR, Akhverdiyeva L, Kuo YF, Volpi E. Developing a screening tool for sarcopenia in hospitalized geriatric patients: Estimation of appendicular skeletal muscle mass using bioelectrical impedance. Clin Nutr. 2020;39(7):2233-7.
46. de Hoogt PA, Reisinger KW, Tegels JJW, Bosmans JWAM, Tijssen F, Stoot JHMB. Functional Compromise Cohort Study (FCCS): Sarcopenia is a Strong Predictor of Mortality in the Intensive Care Unit. World journal of surgery. 2018;42(6):1733-41.
47. Dovjak P. Sarcopenia in cases of chronic and acute illness A mini-review. Zeitschrift Fur Gerontologie Und Geriatrie. 2016;49(2):100-6.
48. Du Y, Karvellas CJ, Baracos V, Williams DC, Khadaroo RG, Group ESA. Sarcopenia is a predictor of outcomes in very elderly patients undergoing emergency surgery. Surgery. 2014;156(3):521-7.
49. Dusseaux MM, Antoun S, Grigioni S, Beduneau G, Carpentier D, Girault C, et al. Skeletal muscle mass and adipose tissue alteration in critically ill patients. Plos One. 2019;14(6).
50. Echeverria I, Besga A, Sanz B, Amasene M, Hervás G, Barroso J, et al. Identification of frailty and sarcopenia in hospitalised older people. European journal of clinical investigation. 2020:e13420.
51. Eglseer D, Hoedl M, Schoberer D. Malnutrition risk and hospital-acquired falls in older adults: A cross-sectional, multicenter study. Geriatrics & Gerontology International. 2020;20(4):348-53.
52. Erbas Sacar D, Kılıc C, Oren MM, Erdogan T, Ozkok S, Ozer Aydın C, et al. Probable sarcopenia: associations with common geriatric syndromes and comorbidities in Turkish geriatric patients from a university hospital. Eur Geriatr Med. 2022;13(6):1299-308.
53. Ferrari U, Schmidmaier R, Jung T, Reincke M, Martini S, Schoser B, et al. IGF-I/IGFBP3/ALS Deficiency in Sarcopenia: Low GHBP Suggests GH Resistance in a Subgroup of Geriatric Patients. J Clin Endocrinol Metab. 2021;106(4):e1698-e707.
54. Fırat Ozer F, Akın S, Soysal T, Gokcekuyu BM, Erturk Zararsız G. Relationship Between Dysphagia and Sarcopenia with Comprehensive Geriatric Evaluation. Dysphagia. 2021;36(1):140-6.
55. Francisco DdS, Martinez L, Terrazas AC, Ribeiro DB, Yamaguti WP. Six-minute stepper test in hospitalized elderly patients: Convergent validity, test-retest reliability and safety. PloS one. 2020;15(10):e0241372.
56. Fu X, Tian Z, Wen S, Sun H, Thapa S, Xiong H, et al. A new index based on serum creatinine and cystatin C is useful for assessing sarcopenia in patients with advanced cancer. Nutrition (Burbank, Los Angeles County, Calif). 2021;82:111032.
57. Fuchs G, Thevathasan T, Chretien YR, Mario J, Piriyapatsom A, Schmidt U, et al. Lumbar skeletal muscle index derived from routine computed tomography exams predict adverse post-extubation outcomes in critically ill patients. Journal of Critical Care. 2018;44:117-23.
58. Gade J, Beck AM, Ronholt F, Andersen HE, Munk T, Vinther A. Validation of the Danish SARC-F in Hospitalized, Geriatric Medical Patients. Journal of Nutrition Health & Aging. 2020;24(10):1120-7.
59. Gade J, Astrup A, Vinther A, Zerahn B. Comparison of a dual-frequency bio-impedance analyser with dual-energy X-ray absorptiometry for assessment of body composition in geriatric patients. Clinical physiology and functional imaging. 2020;40(4):290-301.
60. Gade J, Quick AA, Beck AM, Rønholt F, Vinther A. SARC-F in hospitalized, geriatric medical patients - Feasibility, prevalence of risk of sarcopenia, and characteristics of the risk group, including one-year follow-up. Clinical nutrition ESPEN. 2020;37:80-6.
61. Gariballa S, Alessa A. Sarcopenia: prevalence and prognostic significance in hospitalized patients. Clinical nutrition (Edinburgh, Scotland). 2013;32(5):772-6.
62. Gariballa S, Alessa A. Association between nutritional blood-based biomarkers and clinical outcome in sarcopenia patients. Clinical nutrition ESPEN. 2018;25:145-8.
63. Gariballa S, Alessa A. Cognitive function, depression symptoms and quality of life in sarcopenia patients during both acute illness and recovery. Age and ageing. 2018;47:iii19‐.
64. Geritz J, Maetzold S, Steffen M, Pilotto A, Corrà MF, Moscovich M, et al. Motor, cognitive and mobility deficits in 1000 geriatric patients: protocol of a quantitative observational study before and after routine clinical geriatric treatment - the ComOn-study. BMC geriatrics. 2020;20(1):45.
65. Giannotti C, Nencioni A, Odetti P, Monacelli F. Medication management ability in older patients: time for a reappraisal. Journal of Gerontology and Geriatrics. 2019;67(1):8-12.
66. Gingrich A, Volkert D, Kiesswetter E, Thomanek M, Bach S, Sieber CC, et al. Prevalence and overlap of sarcopenia, frailty, cachexia and malnutrition in older medical inpatients. BMC geriatrics. 2019;19(1):120.
67. Giua R, Pedone C, Scarlata S, Carrozzo I, Rossi FF, Valiani V, et al. Relationship Between Respiratory Muscle Strength and Physical Performance in Elderly Hospitalized Patients. Rejuvenation Research. 2014;17(4):366-71.
68. Goates S, Du K, Arensberg MB, Gaillard T, Guralnik J, Pereira SL. Economic Impact of Hospitalizations in US Adults with Sarcopenia. J Frailty Aging. 2019;8(2):93-9.
69. Gong G, Wan W, Zhang X, Liu Y, Liu X, Yin J. Correlation between the Charlson comorbidity index and skeletal muscle mass/physical performance in hospitalized older people potentially suffering from sarcopenia. BMC geriatrics. 2019;19(1):367.
70. Hamidi M, Ho C, Zeeshan M, O'Keeffe T, Hamza A, Kulvatunyou N, et al. Can Sarcopenia Quantified by Computed Tomography Scan Predict Adverse Outcomes in Emergency General Surgery? The Journal of surgical research. 2019;235:141-7.
71. Hao Q, Hu X, Xie L, Chen J, Jiang J, Dong B, et al. Prevalence of sarcopenia and associated factors in hospitalised older patients: A cross‐sectional study. Australasian journal on ageing. 2018;37(1):62-7.
72. Hartley P, Romero-Ortuno R, Wellwood I, Deaton C. Changes in muscle strength and physical function in older patients during and after hospitalisation: a prospective repeated-measures cohort study. Age and Ageing. 2021;50(1):153-60.
73. Heard R, Black D, Ramsay G, Scott N, Hildebrand D. The prevalence of sarcopaenia in a vascular surgical patient cohort and its impact on outcome. Surgeon (Elsevier Science). 2018;16(6):325-32.
74. Heo WS, Baik HW, Kang JH, Park JS, Park SJ, Jang EJ, et al. The Prevalence of Sarcopenia in Korean Hospitalized Elderly. Annals of Geriatric Medicine and Research. 2015;19(4):235-40.
75. Hernández‐Luis R, Martín‐Ponce E, Monereo‐Muñoz M, Quintero‐Platt G, Odeh‐Santana S, González‐Reimers E, et al. Prognostic value of physical function tests and muscle mass in elderly hospitalized patients. A prospective observational study. Geriatrics & Gerontology International. 2018;18(1):57-64.
76. Hilal S, Perna S, Gasparri C, Alalwan TA, Vecchio V, Fossari F, et al. Comparison between Appendicular Skeletal Muscle Index DXA Defined by EWGSOP1 and 2 versus BIA Tengvall Criteria among Older People Admitted to the Post-Acute Geriatric Care Unit in Italy. Nutrients. 2020;12(6):1818.
77. Hirose S, Nakajima T, Nozawa N, Katayanagi S, Ishizaka H, Mizushima Y, et al. Phase Angle as an Indicator of Sarcopenia, Malnutrition, and Cachexia in Inpatients with Cardiovascular Diseases. Journal of clinical medicine. 2020;9(8).
78. Hu X, Zhang L, Wang H, Hao Q, Dong B, Yang M. Malnutrition-sarcopenia syndrome predicts mortality in hospitalized older patients. Scientific reports. 2017;7(1):1-9.
79. Hu F-J, Liu H, Liu X-L, Jia S-L, Hou L-S, Xia X, et al. Mid-Upper Arm Circumference as an Alternative Screening Instrument to Appendicular Skeletal Muscle Mass Index for Diagnosing Sarcopenia. Clinical interventions in aging. 2021;16:1095-104.
80. Hwang F, McGreevy CM, Pentakota SR, Verde D, Park JH, Berlin A, et al. Sarcopenia is Predictive of Functional Outcomes in Older Trauma Patients. Cureus. 2019;11(11):e6154.
81. Ibrahim K, Howson FFA, Culliford DJ, Sayer AA, Roberts HC. The feasibility of assessing frailty and sarcopenia in hospitalised older people: a comparison of commonly used tools. BMC Geriatrics. 2019;19(1):1-6.
82. Ishida Y, Maeda K, Nonogaki T, Shimizu A, Yamanaka Y, Matsuyama R, et al. SARC-F as a screening tool for sarcopenia and possible sarcopenia proposed by AWGS 2019 in hospitalized older adults. The journal of nutrition, health & aging. 2020;24:1053-60.
83. Ishiyama D, Yamada M, Shikenbaru T, Iwasaki S, Otobe Y, Nishio N, et al. Influence of Physical Characteristics on Readmission in Older Cardiac Patients. Aging Medicine and Healthcare. 2019;10(2):80-7.
84. Jacobsen EL, Brovold T, Bergland A, Bye A. Prevalence of factors associated with malnutrition among acute geriatric patients in Norway: a cross-sectional study. BMJ open. 2016;6(9):e011512.
85. Jang A, Bae CH, Han SJ, Bae H. Association Between Length of Stay in the Intensive Care Unit and Sarcopenia Among Hemiplegic Stroke Patients. Annals of Rehabilitation Medicine-Arm. 2021;45(1):49-56.
86. Ji H-M, Han J, Jin DS, Suh H, Chung Y-S, Won Y-Y. Sarcopenia and Sarcopenic Obesity in Patients Undergoing Orthopedic Surgery. Clinics in orthopedic surgery. 2016;8(2):194-202.
87. Ji W, Liu XL, Zheng KW, Yang HM, Cui JW, Li W. Correlation of phase angle with sarcopenia and its diagnostic value in elderly men with cancer. Nutrition. 2021;84.
88. Jiang Q, Wang Y, Liu Y, Zhu D, Xie Y, Zhao J, et al. Prevalence and associated factors of dry skin among older inpatients in hospitals and nursing homes: A multicenter cross-sectional study. International Journal of Nursing Studies. 2022;135:N.PAG-N.PAG.
89. Joyce PR, O'Dempsey R, Kirby G, Anstey C. A retrospective observational study of sarcopenia and outcomes in critically ill patients. Anaesthesia & Intensive Care. 2020;48(3):229-35.
90. Kaplan SJ, Pham TN, Arbabi S, Gross JA, Damodarasamy M, Bentov I, et al. Association of Radiologic Indicators of Frailty With 1-Year Mortality in Older Trauma Patients: Opportunistic Screening for Sarcopenia and Osteopenia. JAMA Surgery. 2017;152(2):1-8.
91. Khan J, Bath K, Hafeez F, Kim G, Pesola GR. Creatinine Excretion as a Determinant of Accelerated Skeletal Muscle Loss with Critical Illness. Turkish journal of anaesthesiology and reanimation. 2018;46(4):311-5.
92. Kilic MK, Kizilarslanoglu MC, Arik G, Bolayir B, Kara O, Dogan Varan H, et al. Association of Bioelectrical Impedance Analysis-Derived Phase Angle and Sarcopenia in Older Adults. Nutr Clin Pract. 2017;32(1):103-9.
93. Kim Y-S, Lee Y, Chung Y-S, Lee D-J, Joo N-S, Hong D, et al. Prevalence of sarcopenia and sarcopenic obesity in the Korean population based on the Fourth Korean National Health and Nutritional Examination Surveys. Journals of Gerontology Series A: Biomedical Sciences and Medical Sciences. 2012;67(10):1107-13.
94. Kim J-W, Yoon JS, Kim EJ, Hong H-L, Kwon HH, Jung CY, et al. Prognostic Implication of Baseline Sarcopenia for Length of Hospital Stay and Survival in Patients With Coronavirus Disease 2019. The journals of gerontology Series A, Biological sciences and medical sciences. 2021;76(8):e110-e6.
95. Kirk PS, Friedman JF, Cron DC, Terjimanian MN, Wang SC, Campbell DA, et al. One-year postoperative resource utilization in sarcopenic patients. The Journal of surgical research. 2015;199(1):51-5.
96. Kizilarslanoglu MC, Kilic MK, Dogrul RT, Sumer F, Varan HD, Kuyumcu ME, et al. Sarcopenia and length of hospital stay. Is this a vicious cycle? European journal of clinical nutrition. 2016;70(7):863.
97. Koga Y, Fujita M, Yagi T, Todani M, Nakahara T, Kawamura Y, et al. Early enteral nutrition is associated with reduced in-hospital mortality from sepsis in patients with sarcopenia. Journal of critical care. 2018;47:153-8.
98. Kou H-W, Yeh C-H, Tsai H-I, Hsu C-C, Hsieh Y-C, Chen W-T, et al. Sarcopenia is an effective predictor of difficult-to-wean and mortality among critically ill surgical patients. PloS one. 2019;14(8):e0220699.
99. Lamers S, Degerickx R, Vandewoude M, Perkisas S. The mortality determinants of sarcopenia and comorbidities in hospitalized geriatric patients. Journal of frailty, sarcopenia and falls. 2017;2(4):65-72.
100. Lee W-J, Liu L-K, Peng L-N, Lin M-H, Chen L-K, Group IR. Comparisons of sarcopenia defined by IWGS and EWGSOP criteria among older people: results from the I-Lan longitudinal aging study. Journal of the American medical directors association. 2013;14(7):528. e1-. e7.
101. Ligthart-Melis GC, Luiking YC, Kakourou A, Cederholm T, Maier AB, de van der Schueren MAE. Frailty, Sarcopenia, and Malnutrition Frequently (Co-)occur in Hospitalized Older Adults: A Systematic Review and Meta-analysis. Journal of the American Medical Directors Association. 2020;21(9):1216-28.
102. Liu JYJ, Reijnierse EM, van Ancum JM, Verlaan S, Meskers CGM, Maier AB. Acute inflammation is associated with lower muscle strength, muscle mass and functional dependency in male hospitalised older patients. Plos One. 2019;14(4).
103. Loosen SH, Schulze-Hagen M, Püngel T, Bündgens L, Wirtz T, Kather JN, et al. Skeletal Muscle Composition Predicts Outcome in Critically Ill Patients. Critical care explorations. 2020;2(8):e0171.
104. Lu JL, Ding LY, Xu Q, Zhu S-q, Xu XY, Hua HX, et al. Screening Accuracy of SARC-F for Sarcopenia in the Elderly: A Diagnostic Meta-Analysis. Journal of Nutrition, Health & Aging. 2021;25(2):172-82.
105. Maeda K, Akagi J. Sarcopenia is an independent risk factor of dysphagia in hospitalized older people. Geriatrics & gerontology international. 2016;16(4):515-21.
106. Maeda K, Akagi J. Cognitive impairment is independently associated with definitive and possible sarcopenia in hospitalized older adults: The prevalence and impact of comorbidities. Geriatrics & Gerontology International. 2017;17(7):1048-56.
107. Maeda K, Shamoto H, Wakabayashi H, Akagi J. Sarcopenia Is Highly Prevalent in Older Medical Patients With Mobility Limitation. Nutrition in Clinical Practice. 2017;32(1):110-5.
108. Magnuszewski L, Swietek M, Kasiukiewicz A, Kuprjanowicz B, Baczek J, Wojszel ZB. Health, Functional and Nutritional Determinants of Falls Experienced in the Previous Year-A Cross-Sectional Study in a Geriatric Ward. International Journal of Environmental Research and Public Health. 2020;17(13).
109. Malle O, Amrein K, Dimai HP. Sarcopenia and osteoporosis are not independent predictive factors of hospital length of stay and 6 months mortality rate in critically ill patients. Osteoporosis international. 2017;28:S415‐S6.
110. Marinaki V, Lambrou GI. Public Health issues in hospital management of Sarcopenic patients. Journal of frailty, sarcopenia and falls. 2017;2(3):58-61.
111. Marsh AP, Applegate WB, Guralnik JM, Jack Rejeski W, Church TS, Fielding RA, et al. Hospitalizations During a Physical Activity Intervention in Older Adults at Risk of Mobility Disability: Analyses from the Lifestyle Interventions and Independence for Elders Randomized Clinical Trial. Journal of the American Geriatrics Society. 2016;64(5):933-43.
112. Martinez BP, Batista AKMS, Gomes IB, Olivieri FM, Camelier FWR, Camelier AA. Frequency of sarcopenia and associated factors among hospitalized elderly patients. BMC musculoskeletal disorders. 2015;16:108.
113. Martinez BP, Gomes IB, Oliveira CSd, Ramos IR, Rocha MDM, Forgiarini Júnior LA, et al. Accuracy of the Timed Up and Go test for predicting sarcopenia in elderly hospitalized patients. Clinics (Sao Paulo, Brazil). 2015;70(5):369-72.
114. Matsuo H, Yoshimura Y. Calf circumference is associated with dysphagia in acute-care inpatients. Geriatric Nursing. 2018;39(2):186-90.
115. Matsushita T, Nishioka S, Taguchi S, Yamanouchi A, Okazaki Y, Oishi K, et al. Effect of Improvement in Sarcopenia on Functional and Discharge Outcomes in Stroke Rehabilitation Patients. Nutrients. 2021;13(7):2192-.
116. McCusker A, Khan M, Kulvatunyou N, Zeeshan M, Sakran JV, Hayek H, et al. Sarcopenia defined by a computed tomography estimate of the psoas muscle area does not predict frailty in geriatric trauma patients. American journal of surgery. 2019;218(2):261-5.
117. Mendes RML, Pinho CPS, Santana ND, dos Santos NF. Sarcopenia in elderly hospitalized coronary patients. Revista Chilena De Nutricion. 2019;46(1):12-20.
118. Meskers CGM, Reijnierse EM, Numans ST, Kruizinga RC, Pierik VD, Van Ancum JM, et al. Association of Handgrip Strength and Muscle Mass with Dependency in (Instrumental) Activities of Daily Living in Hospitalized Older Adults -The EMPOWER Study. Journal of Nutrition Health & Aging. 2019;23(3):232-8.
119. Moisey LL, Mourtzakis M, Cotton BA, Premji T, Heyland DK, Wade CE, et al. Skeletal muscle predicts ventilator-free days, ICU-free days, and mortality in elderly ICU patients. Critical Care. 2013;17(3):R206-R.
120. Morikawa Y, Kawakami R, Horii M, Yamamoto Y, Yabuta M, Saito Y. Handgrip Strength Is an Independent Predictor of Cardiovascular Outcomes in Diabetes Mellitus. International heart journal. 2021;62(1):50-6.
121. Mueller N, Murthy S, Tainter CR, Lee J, Riddell K, Fintelmann FJ, et al. Can Sarcopenia Quantified by Ultrasound of the Rectus Femoris Muscle Predict Adverse Outcome of Surgical Intensive Care Unit Patients as well as Frailty? A Prospective, Observational Cohort Study. Annals of surgery. 2016;264(6):1116-24.
122. Muscaritoli M, Lucia S, Molfino A. Sarcopenia in critically ill patients: the new pandemia. Minerva anestesiologica. 2013;79(7):771-7.
123. Nagae M, Umegaki H, Yoshiko A, Fujita K, Komiya H, Watanabe K, et al. Echo intensity is more useful in predicting hospital-associated complications than conventional sarcopenia-related parameters in acute hospitalized older patients. Experimental Gerontology. 2021;150.
124. Nagae M, Umegaki H, Yoshiko A, Fujita K, Komiya H, Watanabe K, et al. Muscle Evaluation and Hospital-Associated Disability in Acute Hospitalized Older Adults. The journal of nutrition, health & aging. 2022;26(7):681-7.
125. Nagae M, Umegaki H, Yoshiko A, Fujita K, Komiya H, Watanabe K, et al. Muscle changes on muscle ultrasound and adverse outcomes in acute hospitalized older adults. Nutrition (Burbank, Los Angeles County, Calif). 2022;102:111698.
126. Nagano A, Nishioka S, Wakabayashi H. Rehabilitation Nutrition for Iatrogenic Sarcopenia and Sarcopenic Dysphagia. Journal of Nutrition, Health & Aging. 2019;23(3):256-65.
127. Nam K, Lee JY, Ko Y, Kim KW, Lee HS, Hong SW, et al. Impact of Sarcopenia on Clinical Course of Inflammatory Bowel Disease in Korea. Dig Dis Sci. 2023;68(6):2165-79.
128. Nazli A, Akyuz EY. Assessment of Nutritional Status of Hospitalised Geriatric Patients and its Relationship with Sarcopenia. Istanbul Medical Journal. 2020;21(4):290-6.
129. Nickels MR, Aitken LM, Barnett AG, Walsham J, King S, Gale NE, et al. Effect of in-bed cycling on acute muscle wasting in critically ill adults: A randomised clinical trial. Journal of critical care. 2020;59:86-93.
130. Norman K, Otten L. Financial impact of sarcopenia or low muscle mass - A short review. Clinical nutrition (Edinburgh, Scotland). 2019;38(4):1489-95.
131. Pan SQ, Li YM, Li XF, Xiong R. Sarcopenia in geriatric patients from the plateau region of Qinghai-Tibet: A cross-sectional study. World Journal of Clinical Cases. 2021;9(19):5092-101.
132. Papadopoulou SK, Tsintavis P, Potsaki G, Papandreou D. Differences in the Prevalence of Sarcopenia in Community-Dwelling, Nursing Home and Hospitalized Individuals. A Systematic Review and Meta-Analysis. Journal of Nutrition, Health & Aging. 2020;24(1):83-90.
133. Peng T-C, Chen W-L, Wu L-W, Chang Y-W, Kao T-W. Sarcopenia and cognitive impairment: A systematic review and meta-analysis. Clinical nutrition (Edinburgh, Scotland). 2020;39(9):2695-701.
134. Pérez-Zepeda MU, Sgaravatti A, Dent E. Sarcopenia and post-hospital outcomes in older adults: A longitudinal study. Archives of Gerontology and Geriatrics. 2017;69:105-9.
135. Perkisas S, De Cock A-M, Verhoeven V, Vandewoude M. Intramuscular Adipose Tissue and the Functional Components of Sarcopenia in Hospitalized Geriatric Patients. Geriatrics (Basel, Switzerland). 2017;2(1).
136. Perna S, Francis MD, Bologna C, Moncaglieri F, Riva A, Morazzoni P, et al. Performance of Edmonton Frail Scale on frailty assessment: its association with multi-dimensional geriatric conditions assessed with specific screening tools. BMC Geriatr. 2017;17(1):2.
137. Peterson SJ, Braunschweig CA. Prevalence of Sarcopenia and Associated Outcomes in the Clinical Setting. Nutrition in Clinical Practice. 2016;31(1):40-8.
138. Pierik VD, Meskers CGM, Van Ancum JM, Numans ST, Verlaan S, Scheerman K, et al. High risk of malnutrition is associated with low muscle mass in older hospitalized patients - a prospective cohort study. BMC Geriatrics. 2017;17:118-.
139. Pinotti E, Montuori M, Borrelli V, Giuffrè M, Angrisani L. Sarcopenia: What a Surgeon Should Know. Obesity surgery. 2020;30(5):2015-20.
140. Pourhassan M, Norman K, Muller MJ, Dziewas R, Wirth R. IMPACT OF SARCOPENIA ON ONE-YEAR MORTALITY AMONG OLDER HOSPITALIZED PATIENTS WITH IMPAIRED MOBILITY. Journal of Frailty & Aging. 2018;7(1):40-6.
141. Rasheedy D, El-Kawaly WH. The accuracy of the Geriatric Nutritional Risk Index in detecting frailty and sarcopenia in hospitalized older adults. Aging Clinical & Experimental Research. 2020;32(12):2469-77.
142. Rasheedy D, El-Kawaly WH. The Cumulative Impact of Sarcopenia, Frailty, Malnutrition, and Cachexia on Other Geriatric Syndromes in Hospitalized Elderly. Electronic Journal of General Medicine. 2021;18(2):1-9.
143. Real GG, Fruhauf IR, Sedrez JHK, Dall'Aqua EJF, Gonzalez MC. Calf Circumference: A Marker of Muscle Mass as a Predictor of Hospital Readmission. Journal of Parenteral and Enteral Nutrition. 2018;42(8):1272-9.
144. Reijnierse EM, Buljan A, Tuttle CSL, van Ancum J, Verlaan S, Meskers CGM, et al. Prevalence of sarcopenia in inpatients 70 years and older using different diagnostic criteria. Nursing open. 2018;6(2):377-83.
145. Reijnierse EM, Verlaan S, Pham VK, Lim WK, Meskers CGM, Maier AB. Lower Skeletal Muscle Mass at Admission Independently Predicts Falls and Mortality 3 Months Post-discharge in Hospitalized Older Patients. Journals of Gerontology Series A: Biological Sciences & Medical Sciences. 2019;74(10):1650-6.
146. Reiss J, Iglseder B, Kreutzer M, Weilbuchner I, Treschnitzer W, Kässmann H, et al. Case finding for sarcopenia in geriatric inpatients: performance of bioimpedance analysis in comparison to dual X-ray absorptiometry. BMC Geriatrics. 2016;16:1-8.
147. Reiss J, Iglseder B, Alzner R, Mayr-Pirker B, Pirich C, Kässmann H, et al. Sarcopenia and osteoporosis are interrelated in geriatric inpatients. Zeitschrift für Gerontologie und Geriatrie. 2019;52(7):688-93.
148. Reiss J, Iglseder B, Alzner R, Mayr-Pirker B, Pirich C, Kassmann H, et al. Consequences of applying the new EWGSOP2 guideline instead of the former EWGSOP guideline for sarcopenia case finding in older patients. Age and Ageing. 2019;48(5):713-8.
149. Rommersbach N, Wirth R, Lueg G, Klimek C, Schnatmann M, Liermann D, et al. The impact of disease-related immobilization on thigh muscle mass and strength in older hospitalized patients. Bmc Geriatrics. 2020;20(1).
150. Rossi AP, Fantin F, Micciolo R, Bertocchi M, Bertassello P, Zanandrea V, et al. Identifying Sarcopenia in Acute Care Setting Patients. Journal of the American Medical Directors Association. 2014;15(4):303.e7-.e12.
151. Rossi AP, Fantin F, Abete P, Bellelli G, Bo M, Cherubini A, et al. Association between hospitalization-related outcomes, dynapenia and body mass index: The Glisten Study. European Journal of Clinical Nutrition. 2019;73(5):743-50.
152. Rus GE, Porter J, Brunton A, Crocker M, Kotsimbos Z, Percic J, et al. Nutrition interventions implemented in hospital to lower risk of sarcopenia in older adults: A systematic review of randomised controlled trials. Nutrition & dietetics: the journal of the Dietitians Association of Australia. 2020;77(1):90-102.
153. Rustani K, Kundisova L, Capecchi PL, Nante N, Bicchi M. Prevalence of sarcopenia and its impact on mortality and readmission rates amongest geriatric patients. Journal of Gerontology and Geriatrics. 2019;67(4):200-6.
154. Sáez de Asteasu ML, Martínez-Velilla N, Zambom-Ferraresi F, Ramírez-Vélez R, García-Hermoso A, Cadore EL, et al. Changes in muscle power after usual care or early structured exercise intervention in acutely hospitalized older adults. Journal of cachexia, sarcopenia and muscle. 2020;11(4):997-1006.
155. Sáez de Asteasu ML, Martínez-Velilla N, Zambom-Ferraresi F, Casas-Herrero Á, Cadore EL, Ramirez-Velez R, et al. Inter-individual variability in response to exercise intervention or usual care in hospitalized older adults. J Cachexia Sarcopenia Muscle. 2019;10(6):1266-75.
156. Safonova JA, Glazunova GM. [Diagnostic criteria and the prevalence of sarcopenia in the elderly.]. Advances in gerontology = Uspekhi gerontologii. 2019;32(6):882-8.
157. Sanchez-Rodriguez D, Marco E, Miralles R, Fayos M, Mojal S, Alvarado M, et al. Sarcopenia, physical rehabilitation and functional outcomes of patients in a subacute geriatric care unit. Archives of Gerontology and Geriatrics. 2014;59(1):39-43.
158. Sánchez-Rodríguez D, Marco E, Ronquillo-Moreno N, Miralles R, Vázquez-Ibar O, Escalada F, et al. Prevalence of malnutrition and sarcopenia in a post-acute care geriatric unit: Applying the new ESPEN definition and EWGSOP criteria. Clinical Nutrition. 2017;36(5):1339-44.
159. Sánchez-Torralvo FJ, Ruiz-García I, Contreras-Bolívar V, González-Almendros I, Ruiz-Vico M, Abuín-Fernández J, et al. CT-Determined Sarcopenia in GLIM-Defined Malnutrition and Prediction of 6-Month Mortality in Cancer Inpatients. Nutrients. 2021;13(8).
160. Savas S, Yilmaz M. Self Reported Dysphagia is not Associated with Sarcopenia Defined by the Revised EWGSOP2 Criteria and Regional Thresholds at the Hospital Among Ambulatory Older Patients. Materia socio-medica. 2019;31(4):253-7.
161. Scheerman K, Meskers CGM, Verlaan S, Maier AB. Sarcopenia, Low Handgrip Strength, and Low Absolute Muscle Mass Predict Long-Term Mortality in Older Hospitalized Patients: An Observational Inception Cohort Study. Journal of the American Medical Directors Association. 2021.
162. Shibahashi K, Sugiyama K, Kashiura M, Hamabe Y. Decreasing skeletal muscle as a risk factor for mortality in elderly patients with sepsis: a retrospective cohort study. Journal of intensive care. 2017;5:8.
163. Sipers WM, Meijers JM, van Dijk RB, Halfens RJ, Schols JM. Impact of Different Diagnostic Criteria on the Prevalence of Sarcopenia in an Acute Care Geriatric Ward. J Frailty Aging. 2014;3(4):222-9.
164. Sipers WMWH, de Blois W, Schols JMGA, van Loon LJC, Verdijk LB. Sarcopenia is Related to Mortality in the Acutely Hospitalized Geriatric Patient. Journal of Nutrition, Health & Aging. 2019;23(2):128-37.
165. Sipers WMWH, Dorge J, Schols JMGA, Verdijk LB, van Loon LJC. Multifrequency bioelectrical impedance analysis may represent a reproducible and practical tool to assess skeletal muscle mass in euvolemic acutely ill hospitalized geriatric patients. European geriatric medicine. 2020;11(1):155-62.
166. Slee A, Birch D, Stokoe D. A comparison of the malnutrition screening tools, MUST, MNA and bioelectrical impedance assessment in frail older hospital patients. Clinical Nutrition. 2015;34(2):296-301.
167. Smithard D, Hansjee D, Henry D, Mitchell L, Sabaharwal A, Salkeld J, et al. Inter-Relationships between Frailty, Sarcopenia, Undernutrition and Dysphagia in Older People Who Are Admitted to Acute Frailty and Medical Wards: Is There an Older Adult Quartet? Geriatrics (Basel, Switzerland). 2020;5(3).
168. Smoliner C, Sieber CC, Wirth R. Prevalence of Sarcopenia in Geriatric Hospitalized Patients. Journal of the American Medical Directors Association. 2014;15(4):267-72.
169. Sobestiansky S, Åberg AC, Cederholm T. Sarcopenia and malnutrition in relation to mortality in hospitalised patients in geriatric care - predictive validity of updated diagnoses. Clin Nutr ESPEN. 2021;45:442-8.
170. Sousa AS, Guerra RS, Fonseca I, Pichel F, Amaral TF. Sarcopenia among hospitalized patients–a cross-sectional study. Clinical nutrition. 2015;34(6):1239-44.
171. Sousa AS, Guerra RS, Fonseca I, Pichel F, Amaral TF. Sarcopenia and length of hospital stay. European journal of clinical nutrition. 2016;70(5):595-601.
172. Sousa IM, Burgel CF, Silva FM, Fayh APT. Prognostic Value of Isolated Sarcopenia or Malnutrition-Sarcopenia Syndrome for Clinical Outcomes in Hospitalized Patients. Nutrients. 2022;14(11).
173. Spahillari A, Mukamal KJ, DeFilippi C, Kizer JR, Gottdiener JS, Djoussé L, et al. The association of lean and fat mass with all-cause mortality in older adults: The Cardiovascular Health Study. Nutrition, metabolism, and cardiovascular diseases : NMCD. 2016;26(11):1039-47.
174. Sperlich E, Fleiner T, Zijlstra W, Haussermann P, Morat T. Sarcopenia in geriatric psychiatry: feasibility of the diagnostic process and estimation of prevalence within a hospital context. Journal of Cachexia Sarcopenia and Muscle. 2021;12(5):1153-60.
175. Stuck AK, Mäder NC, Bertschi D, Limacher A, Kressig RW. Performance of the EWGSOP2 Cut-Points of Low Grip Strength for Identifying Sarcopenia and Frailty Phenotype: A Cross-Sectional Study in Older Inpatients. International journal of environmental research and public health. 2021;18(7).
176. Surkan MJ, Gibson W. Interventions to Mobilize Elderly Patients and Reduce Length of Hospital Stay. The Canadian journal of cardiology. 2018;34(7):881-8.
177. Tang T, Wu L, Yang L, Jiang J, Hao Q, Dong B, et al. A sarcopenia screening test predicts mortality in hospitalized older adults. Scientific reports. 2018;8(1):2923.
178. Tao J, Ke Y-Y, Zhang Z, Zhang Y, Wang Y-Y, Ren C-X, et al. Comparison of the value of malnutrition and sarcopenia for predicting mortality in hospitalized old adults over 80 years. Experimental gerontology. 2020;138:111007.
179. Tatsumi M, Kumagai S, Abe T, Murakami S, Takeda H, Shichinohe T, et al. Sarcopenia in a patient with most serious complications after highly invasive surgeries treated with nutrition, rehabilitation, and pharmacotherapy: a case report. Journal of Pharmaceutical Health Care and Sciences. 2021;7(1).
180. Teschler M, Heimer M, Schmitz B, Kemmler W, Mooren FC. Four weeks of electromyostimulation improves muscle function and strength in sarcopenic patients: a three-arm parallel randomized trial. Journal of cachexia, sarcopenia and muscle. 2021.
181. Thomas DR. Sarcopenia. Clinics in geriatric medicine. 2010;26(2):331-46.
182. Toptas M, Yalcin M, Akkoc İ, Demir E, Metin C, Savas Y, et al. The Relation between Sarcopenia and Mortality in Patients at Intensive Care Unit. BioMed research international. 2018;2018:5263208.
183. Trethewey SP, Brown N, Gao F, Turner AM. Interventions for the management and prevention of sarcopenia in the critically ill: A systematic review. Journal of critical care. 2019;50:287-95.
184. Tsekoura M, Kastrinis A, Katsoulaki M, Billis E, Gliatis J. Sarcopenia and Its Impact on Quality of Life. Advances in experimental medicine and biology. 2017;987:213-8.
185. Van Ancum JM, Scheerman K, Pierik VD, Numans ST, Verlaan S, Smeenk HE, et al. Muscle Strength and Muscle Mass in Older Patients during Hospitalization: The EMPOWER Study. Gerontology. 2017;63(6):507-14.
186. Van Ancum JM, Pijnappels M, Jonkman NH, Scheerman K, Verlaan S, Meskers CGM, et al. Muscle mass and muscle strength are associated with pre- and post-hospitalization falls in older male inpatients: a longitudinal cohort study. BMC Geriatrics. 2018;18(1):1-7.
187. Van Ancum JM, Alcazar J, Meskers CGM, Nielsen BR, Suetta C, Maier AB. Impact of using the updated EWGSOP2 definition in diagnosing sarcopenia: A clinical perspective. Archives of Gerontology & Geriatrics. 2020;90:N.PAG-N.PAG.
188. van Dam R, van Ancum JM, Verlaan S, Scheerman K, Meskers CGM, Maier AB. Lower Cognitive Function in Older Patients with Lower Muscle Strength and Muscle Mass. Dementia and Geriatric Cognitive Disorders. 2018;45(3-4):243-50.
189. van Dronkelaar C, Tieland M, Aarden JJ, Reichardt LA, van Seben R, van der Schaaf M, et al. Decreased Appetite is Associated with Sarcopenia-Related Outcomes in Acute Hospitalized Older Adults. Nutrients. 2019;11(4):932.
190. Van Nguyen T, Tran KD, Bui KX, Le D, Nguyen TN. A preliminary study to identify the likely risk for sarcopenia in older hospitalised patients with cardiovascular disease in Vietnam. Australasian Journal on Ageing. 2020;39(3):e315-e21.
191. Vetrano DL, Landi F, Volpato S, Corsonello A, Meloni E, Bernabei R, et al. Association of sarcopenia with short-and long-term mortality in older adults admitted to acute care wards: results from the CRIME study. Journals of Gerontology Series A: Biomedical Sciences and Medical Sciences. 2014;69(9):1154-61.
192. Wahlen BM, Mekkodathil A, Al-Thani H, El-Menyar A. Impact of sarcopenia in trauma and surgical patient population: A literature review. Asian journal of surgery. 2020;43(6):647-53.
193. Wang R, Liang Y, Jiang J, Chen M, Li L, Yang H, et al. Effectiveness of a Short-Term Mixed Exercise Program for Treating Sarcopenia in Hospitalized Patients Aged 80 Years and Older: A Prospective Clinical Trial. Journal of Nutrition, Health & Aging. 2020;24(10):1087-93.
194. Wilson DV, Moorey H, Stringer H, Sahbudin I, Filer A, Lord JM, et al. Bilateral Anterior Thigh Thickness: A New Diagnostic Tool for the Identification of Low Muscle Mass? Journal of the American Medical Directors Association. 2019;20(10):1247-.
195. Wollersheim T, Grunow JJ, Carbon NM, Haas K, Malleike J, Ramme SF, et al. Muscle wasting and function after muscle activation and early protocol-based physiotherapy: an explorative trial. J Cachexia Sarcopenia Muscle. 2019;10(4):734-47.
196. Woo HY, Oh S-Y, Lee H, Ryu HG. Evaluation of the association between decreased skeletal muscle mass and extubation failure after long-term mechanical ventilation. Clinical nutrition (Edinburgh, Scotland). 2020;39(9):2764-70.
197. Xu L, Zhang J, Shen S, Hong X, Zeng X, Yang Y, et al. Association Between Body Composition and Frailty in Elder Inpatients. Clinical interventions in aging. 2020;15:313-20.
198. Yao L, Petrosyan A, Fuangfa P, Lenchik L, Boutin RD. Diagnosing sarcopenia at the point of imaging care: analysis of clinical, functional, and opportunistic CT metrics. Skeletal radiology. 2021;50(3):543-50.
199. Yeh DD, Ortiz-Reyes LA, Quraishi SA, Chokengarmwong N, Avery L, Kaafarani HMA, et al. Early nutritional inadequacy is associated with psoas muscle deterioration and worse clinical outcomes in critically ill surgical patients. Journal of critical care. 2018;45:7-13.
200. Yu MD, Zhang HZ, Zhang Y, Yang SP, Lin M, Zhang YM, et al. Relationship between chronic kidney disease and sarcopenia. Scientific Reports. 2021;11(1).
201. Zengarini E, Giacconi R, Mancinelli L, Riccardi GR, Castellani D, Vetrano DL, et al. Prognosis and interplay of cognitive impairment and sarcopenia in older adults discharged from acute care hospitals. Journal of Clinical Medicine. 2019;8(10):1693.
202. Zhang X-L, Zhang Z, Zhu Y-X, Tao J, Zhang Y, Wang Y-Y, et al. Comparison of the efficacy of Nutritional Risk Screening 2002 and Mini Nutritional Assessment Short Form in recognizing sarcopenia and predicting its mortality. European journal of clinical nutrition. 2020;74(7):1029-37.
203. Zhao Y, Zhang Y, Hao Q, Ge M, Dong B. Sarcopenia and hospital-related outcomes in the old people: a systematic review and meta-analysis. Aging Clinical & Experimental Research. 2019;31(1):5-14.
204. Zumsteg DM, Chu CE, Midwinter MJ. Radiographic assessment of sarcopenia in the trauma setting: a systematic review. Trauma surgery & acute care open. 2020;5(1):e000414.
