# Supplementary material for: Incident sarcopenia in hospitalized older people: A systematic review
Source: PLoS One. 2023 Aug 2;18(8):e0289379. doi: 10.1371/journal.pone.0289379 (PMC10395895; doi:10.1371/journal.pone.0289379)
Supplement: S4 Appendix — (DOCX) [file pone.0289379.s004.docx]

**Appendix 4.** **Example of extracted data table**

Table 4: Data extraction form and related detail

| Study details | Year |  |
| --- | --- | --- |
|  | First author |  |
|  | Location (Country) |  |
|  | Health care setting |  |
|  | Diagnostic criteria used |  |
|  | Type of study |  |
| Overall study sample population | Sample size (intervention/control/other group) |  |
|  | Gender (M, F) |  |
|  | Age (report mean (SD), median (range, IQR, and age groups, n%) ) |  |
|  | Clinical area / specialty |  |
| Sarcopenia specific sample population | Number participants |  |
|  | Gender (M, F) |  |
|  | Age (report mean (SD), median (range, IQR, and age groups, n%) ) |  |
|  | Ethnicity (Asian, Caucasian, African, Others) |  |
|  | Frailty |  |
|  | Nutritional status (malnourished, normal) |  |
|  | BMI (underweight, normal/overweight) |  |
|  | Comorbidity (single, multiple), by system ie neurological, endocrine, malignancy, etc  Either by comorbidity cumulative score or diagnosis count  List comorbid condition, n% |  |
|  | Polypharmacy (>4) |  |
| Epidemiology | When diagnosed? |  |
|  | General prevalence |  |
|  | General incidence |  |
| Muscle measurements | Day into admission sarcopenia assessment |  |
|  | Muscle strength test used |  |
|  | Muscle strength reported, mean (SD) or median (range, IQR) |  |
|  | Skeletal muscle mass test used |  |
|  | Skeletal muscle mass reported, mean (SD) or median (range, IQR) |  |
|  | Muscle performance used |  |
|  | Muscle performance reported, mean (SD) or median (range, IQR) |  |
|  | Repeated muscle measurements?  Date repeated, what was repeated and reported change |  |
| Risk factors for sarcopenia | Pre-sarcopenia/at risk for sarcopenia, sarcopenia, severe sarcopenia  List each risk factor and effect size, eg OR, RR with CI and p-value for the development of sarcopenia (yes/no) and severity of sarcopenia (pre-, sarcopenia and severe sarcopenia) |  |
| Outcome measures  List each outcome and effect size (eg compared to non-sarcopenia if available), eg HR. RR with CI and p-value for the development of the specific outcome. List any reported confounding factors. | Mortality (inpatient and post-discharge) |  |
|  | Inpatient complications |  |
|  | Length of hospital stay |  |
|  | Readmission |  |
|  | Reduced function |  |
|  | Reduced mobility |  |
|  | Quality of life (any measure of QoL) |  |
