# Supplementary material for: Incident sarcopenia in hospitalized older people: A systematic review
Source: PLoS One. 2023 Aug 2;18(8):e0289379. doi: 10.1371/journal.pone.0289379 (PMC10395895; doi:10.1371/journal.pone.0289379)

**Appendix 5. Quality assessment table and JBI appraisal forms**

Table 5. Joanna Briggs Institute Checklist for Cohort studies

| **Study/ Domains** | Q1. Were the two groups similar and recruited from the same population? | Q2. Were the exposures measured similarly to assign people to both exposed and  unexposed groups? | Q3. Was the exposure measured in a valid and reliable way? | Q4 Were confounding factors identified? | Q5. Were strategies to deal with confounding factors stated? | Q6. Were the groups/participants free of the outcome at the start of the study (or at the  moment of exposure)? | Q7. Were the outcomes measured in a valid and reliable way? | Q8. Was the follow up time reported and sufficient to be long enough for  outcomes to occur? | Q9. Was follow up complete, and if not, were the reasons to loss to follow up described  and explored? | Q10. Were strategies to address incomplete follow up utilized? | Q11. Was appropriate statistical analysis used? | **Domains completed** |
| --- | --- | --- | --- | --- | --- | --- | --- | --- | --- | --- | --- | --- |
| Aarden et al., 2021  ^(1)^ | U | Y | Y | Y | Y | U | U | Y | Y | Y | Y | 8/11, Moderate |
| Ballesteros-Pomar et al., 2021 ^(2)^ | U | Y | U | U | U | Y | Y | Y | Y | N | Y | 6/11, Moderate |
| Martone et al., 2017 ^(3)^ | N/A | Y | Y | U | U | Y | Y | Y | Y | N | Y | 7/11, Moderate |
| Welch et al., 2022 ^(4)^ | N | Y | Y | Y | U | Y | N | Y | Y | Y | Y | 8/11, Moderate |

Y - Yes, N - No, U - Unclear, N/A - not applicable

Table 6. Joanna Briggs Institute Checklist for Clinical Trials

| **Study/Domain** | Q1. Was true randomization used for assignment of participants to treatment groups? | Q2. Was allocation to groups concealed? | Q3. Were treatment groups similar at the baseline? | Q4. ere participants blind to treatment assignment? | Q5 Were those delivering treatment blind to treatment assignment? | Q6. Were outcomes assessors blind to treatment assignment? | Q7 Were treatment groups treated identically other than the intervention of interest? | Q8 Was follow up complete and if not, were differences between groups in terms of their follow up adequately described and analyzed? | Q9. Were participants analyzed in the groups to which they were randomized? | Q10. Were outcomes measured in the same way for treatment groups? | Q11. Were outcomes measured in a reliable way? | Q12. Was appropriate statistical analysis used? | Q13. Was the trial design appropriate for the topic, and any deviations from the standard RCT  design accounted for in the conduct and analysis? | **Domains completed** |
| --- | --- | --- | --- | --- | --- | --- | --- | --- | --- | --- | --- | --- | --- | --- |
| Gade  et al.,  2019 ^(5)^ | U | U | Y | Y | Y | U | Y | Y | Y | U | Y | Y | N | 8/13,  Mo-  derate |

Y - Yes, N - No, U - Unclear, N/A - not applicable


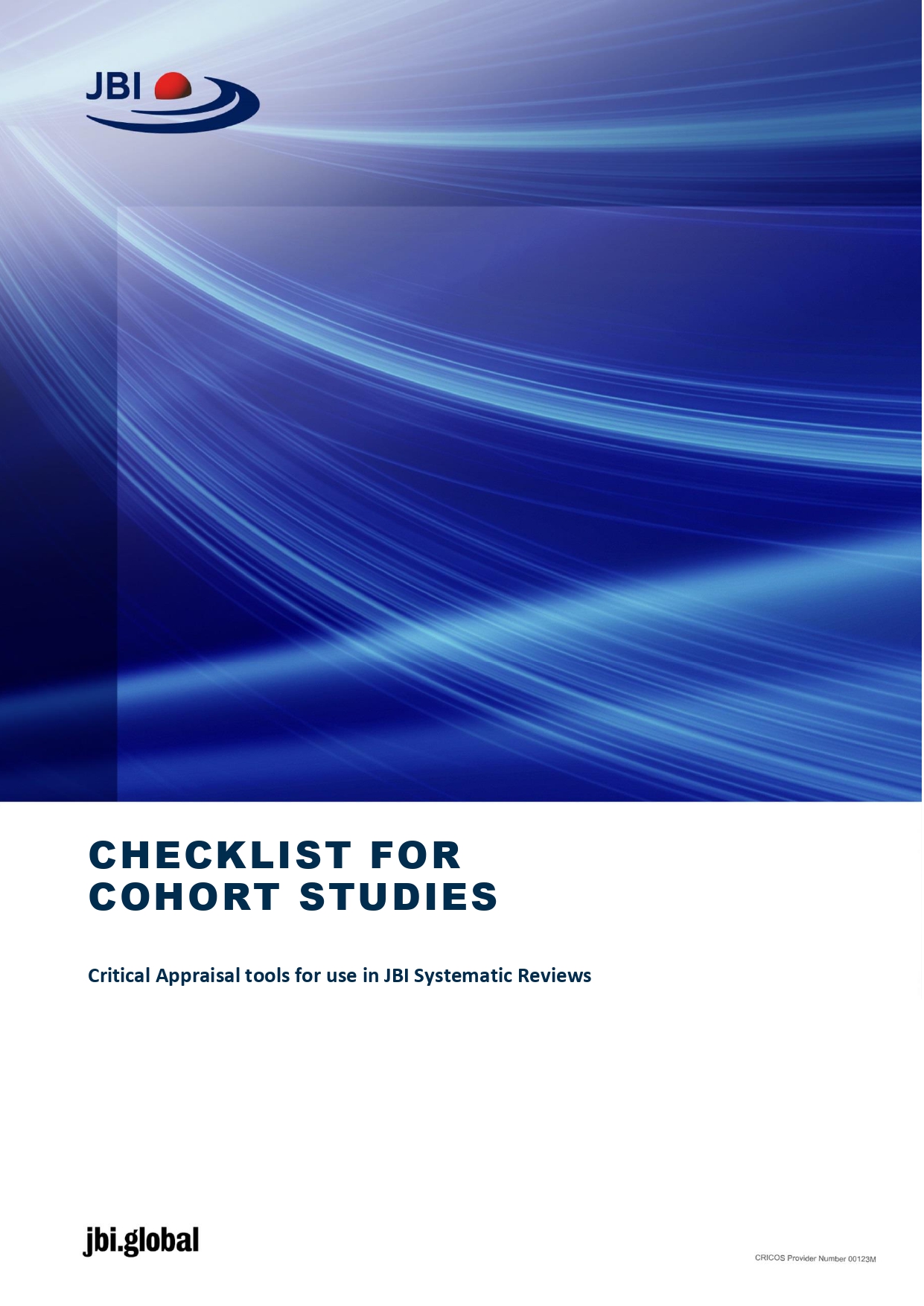

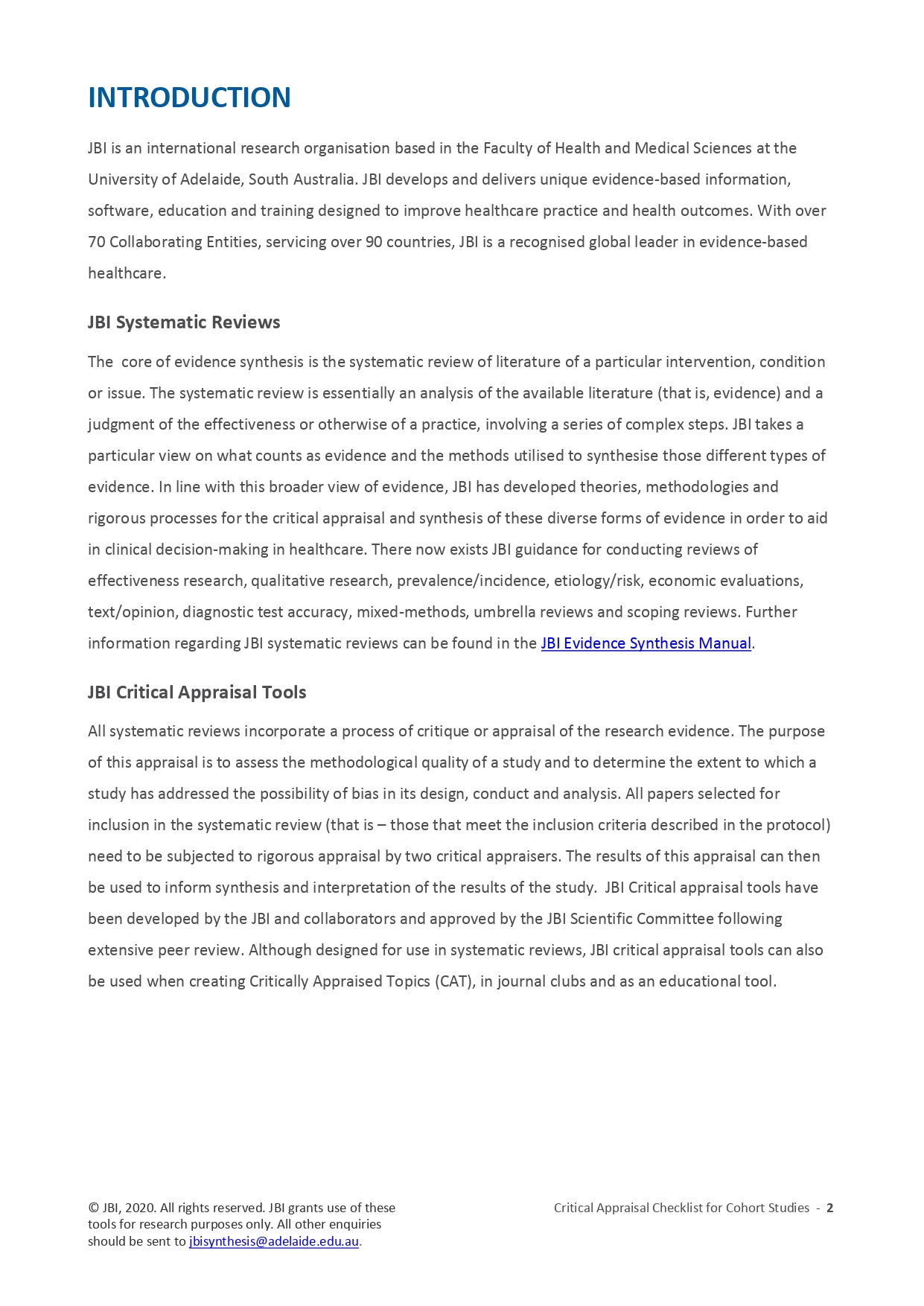

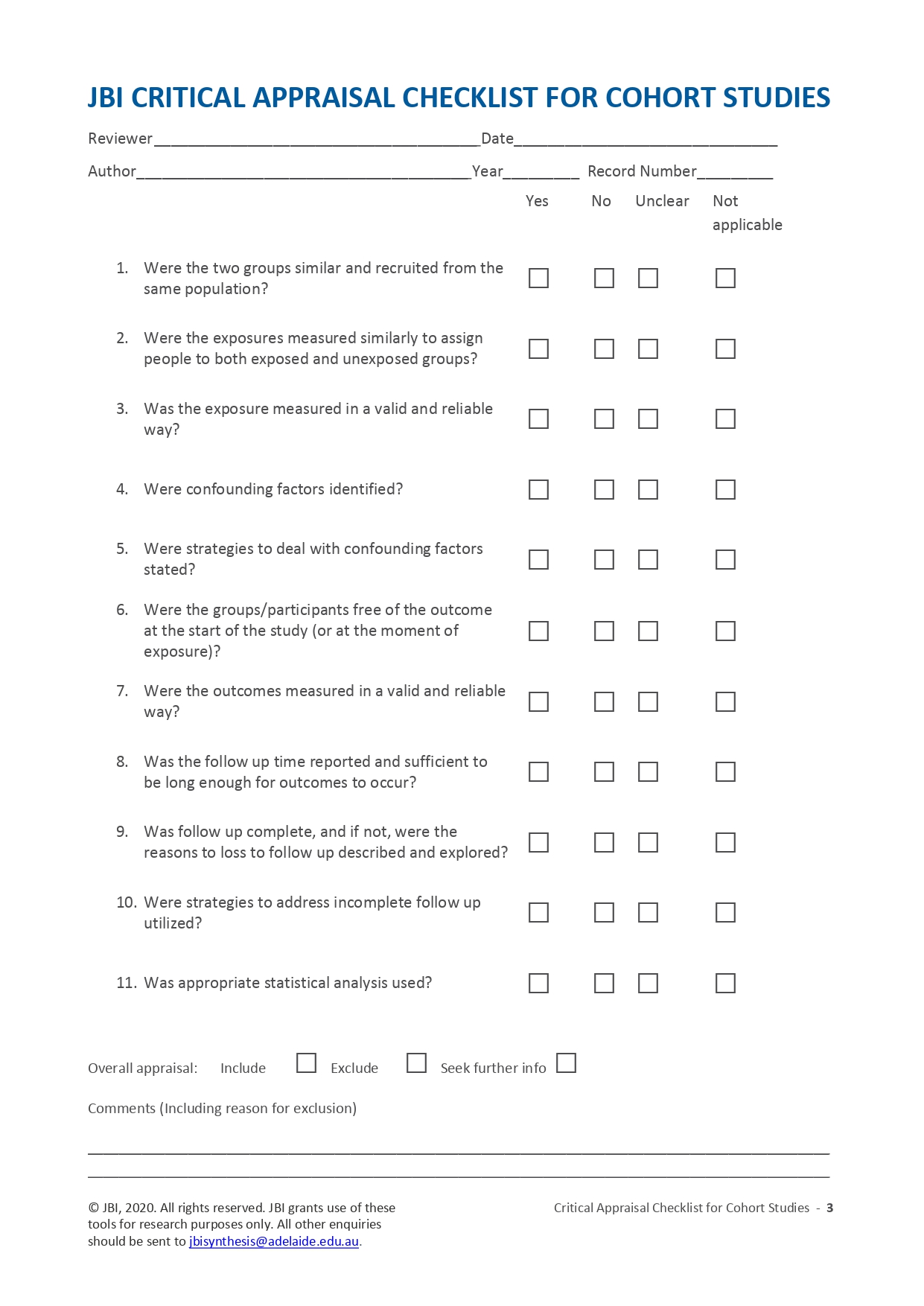

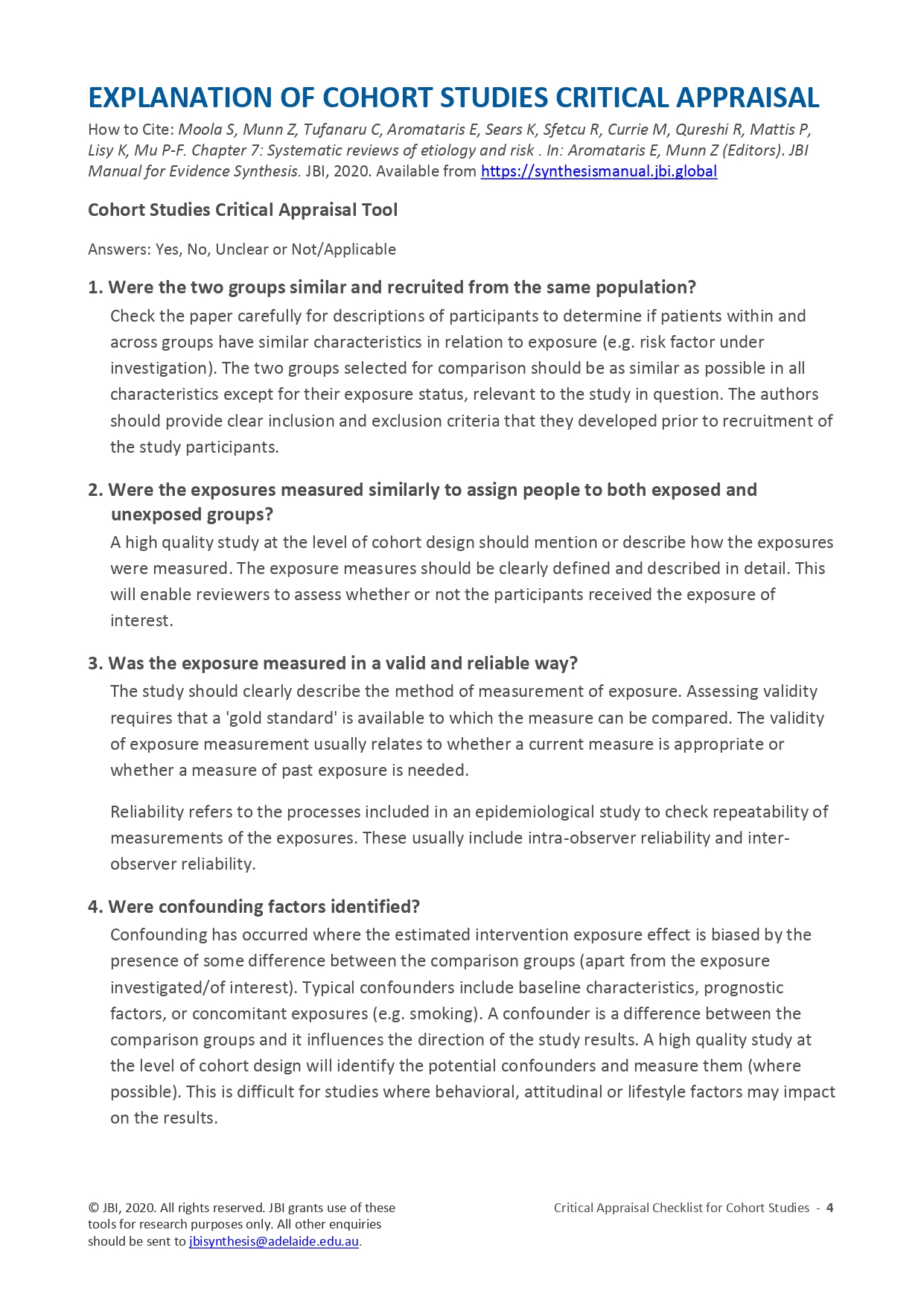

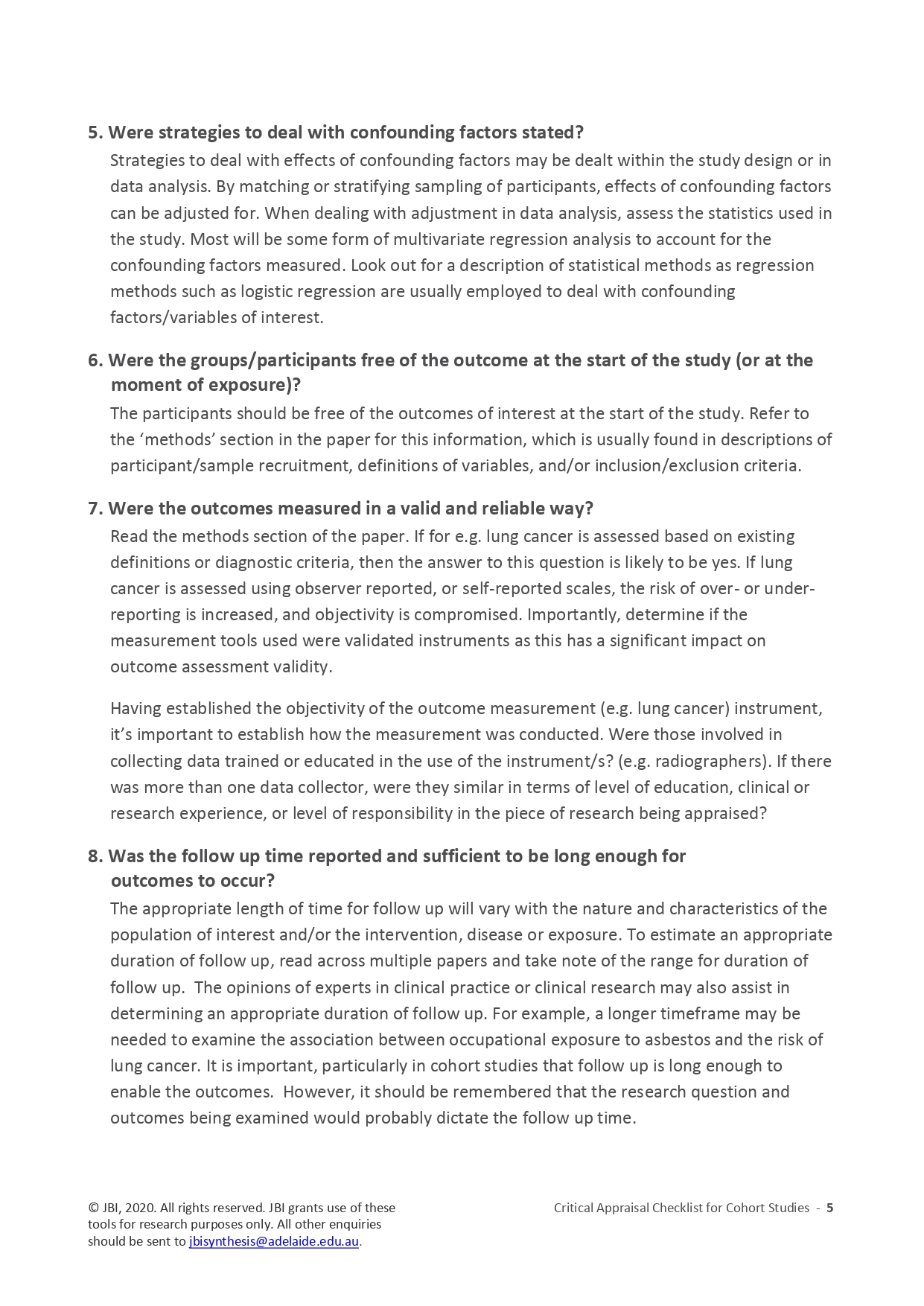

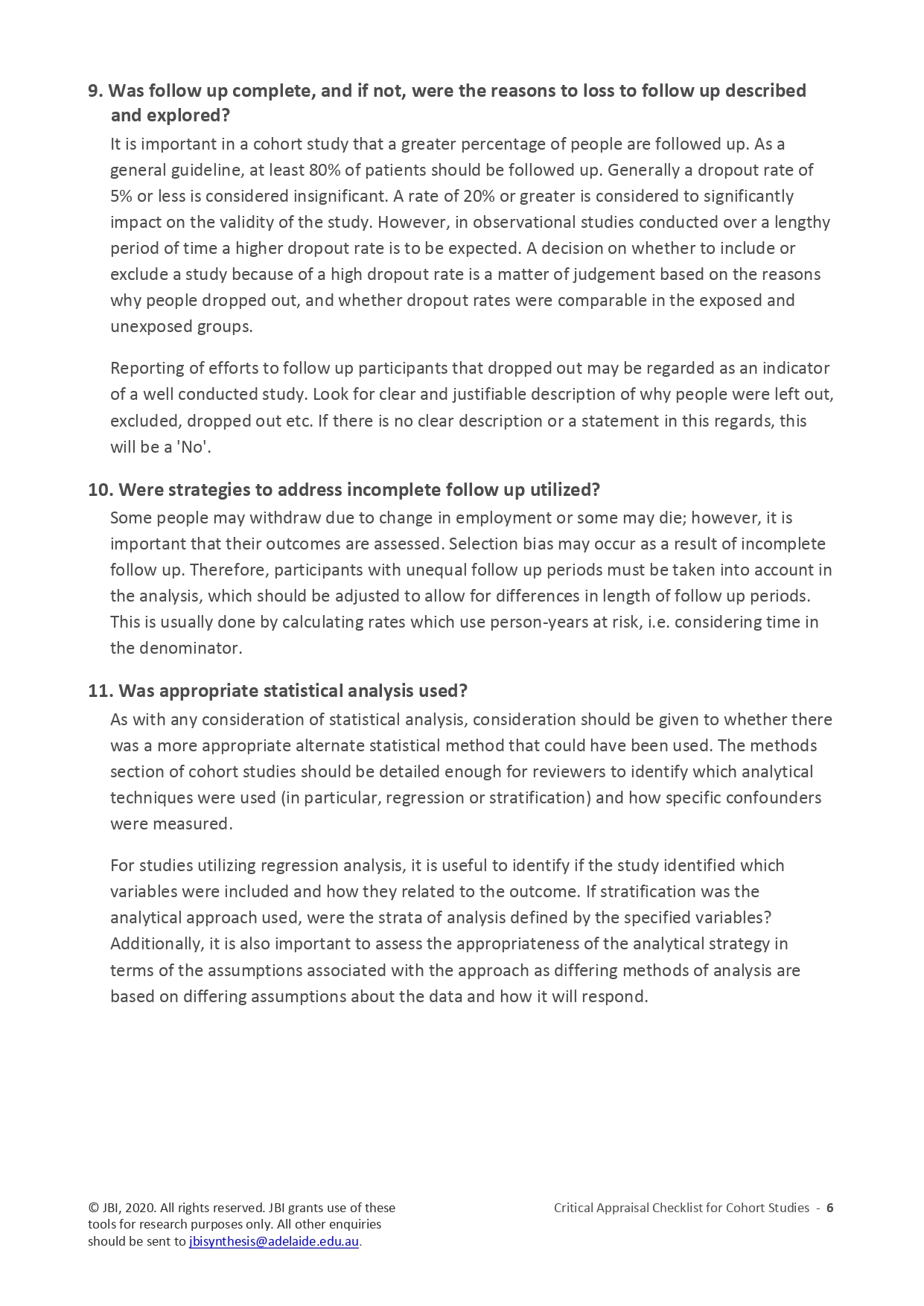


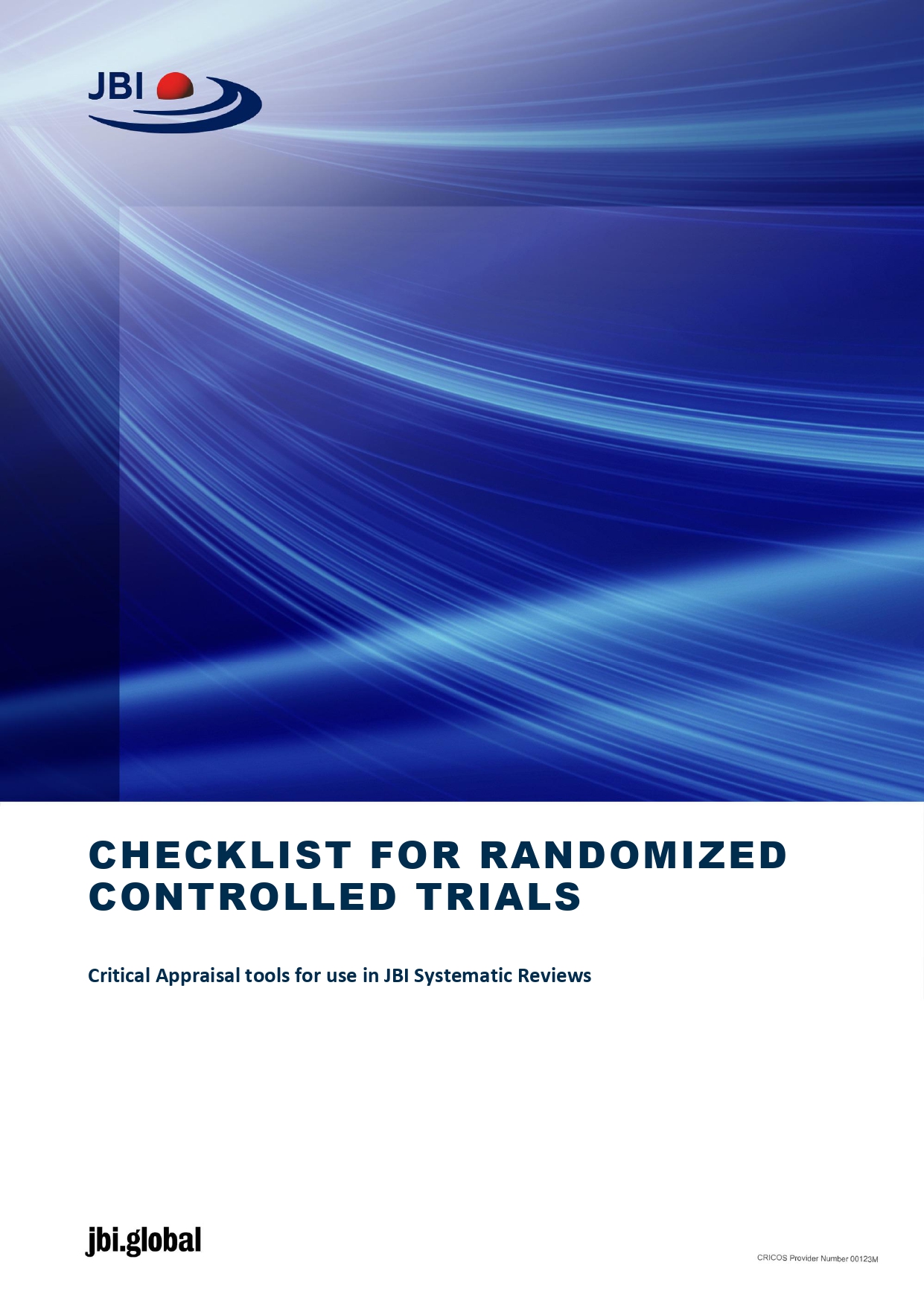

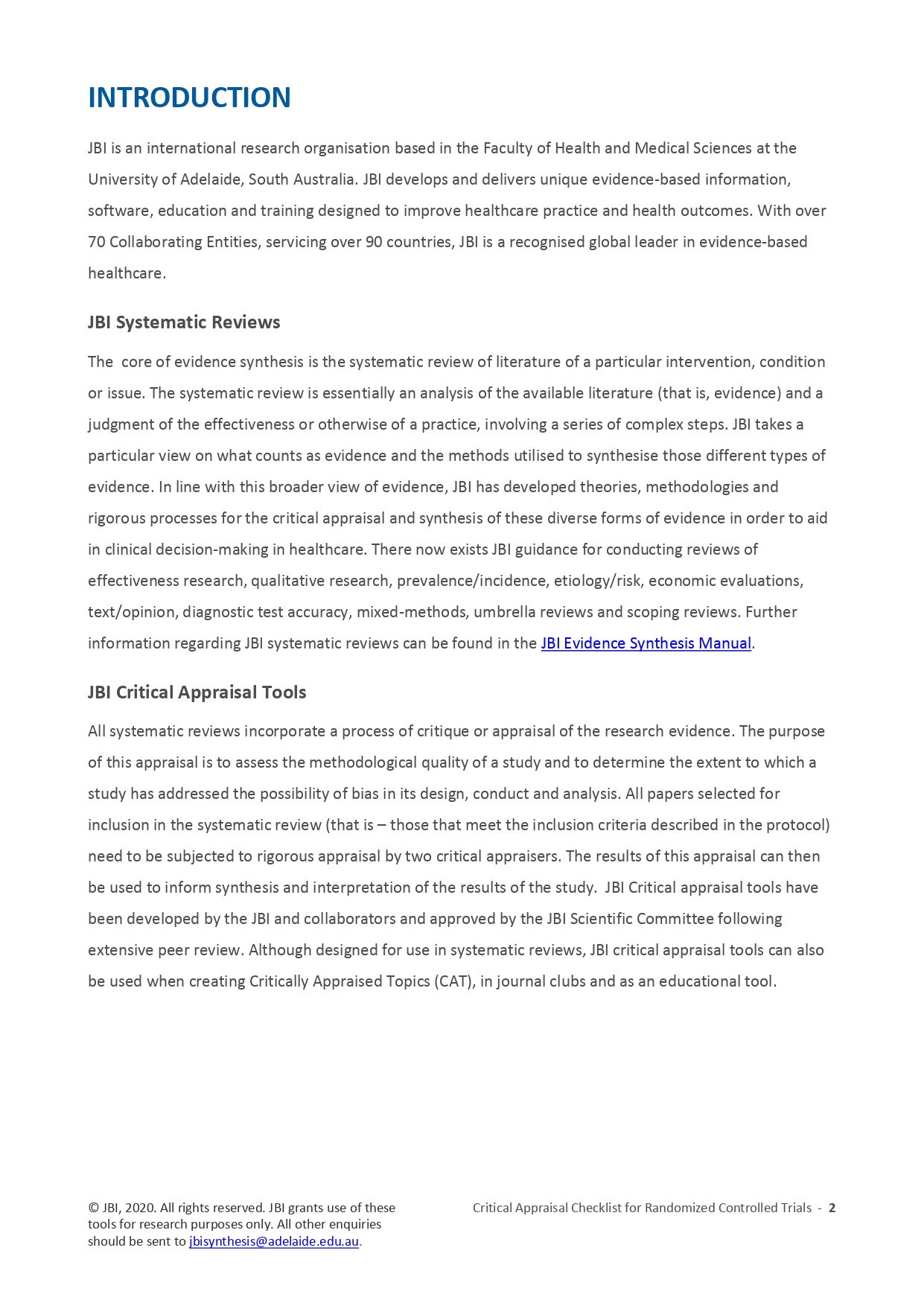

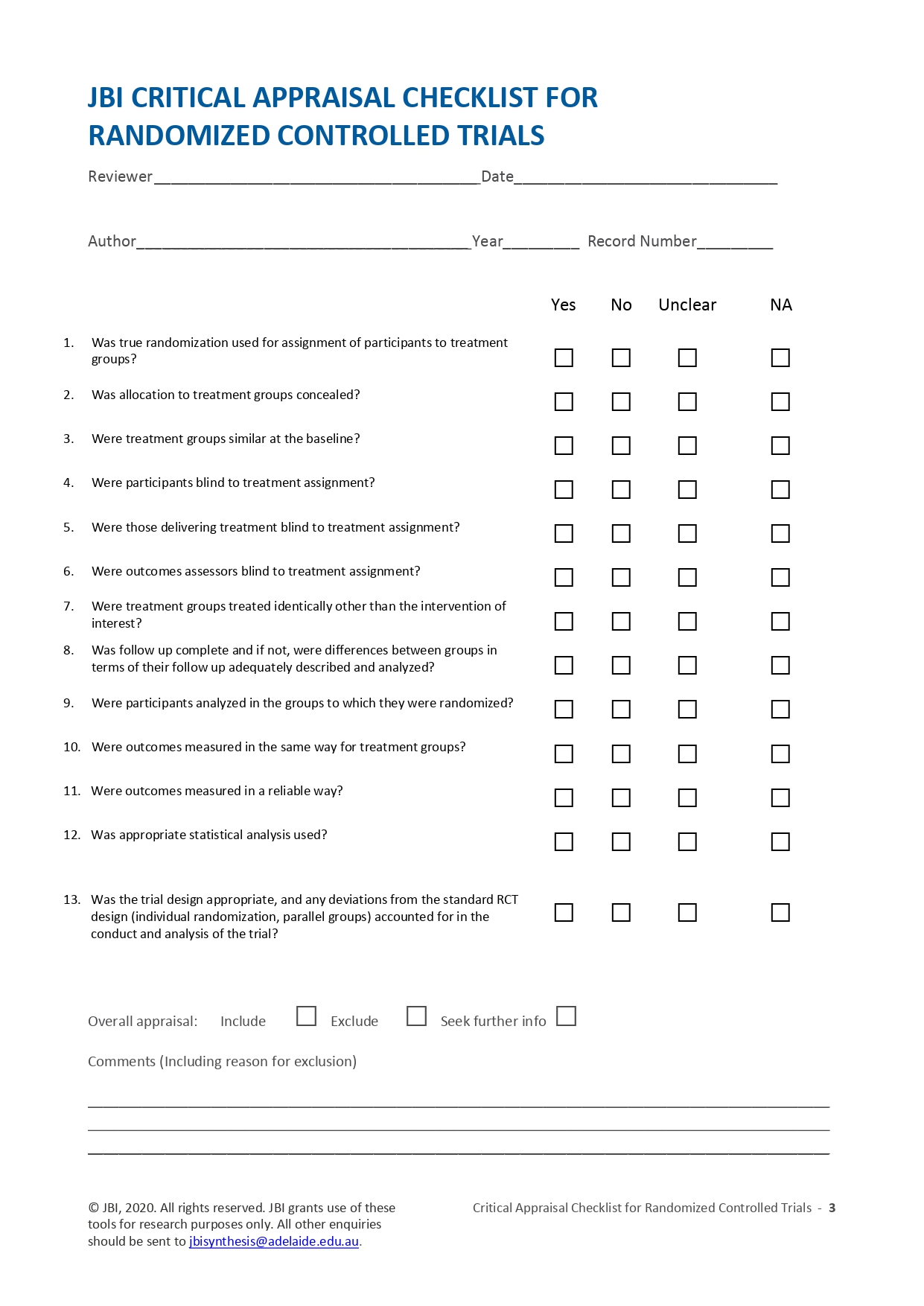

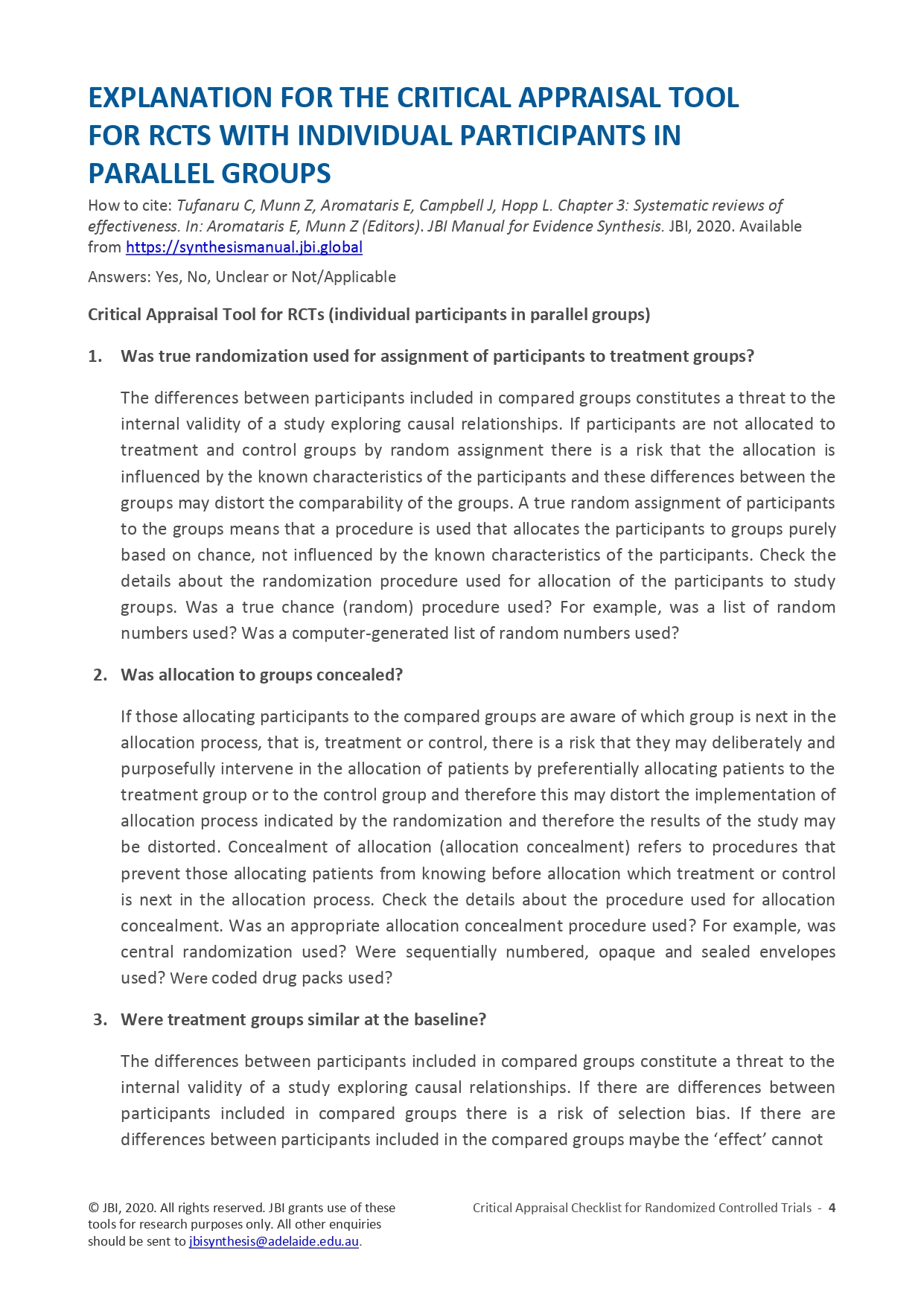

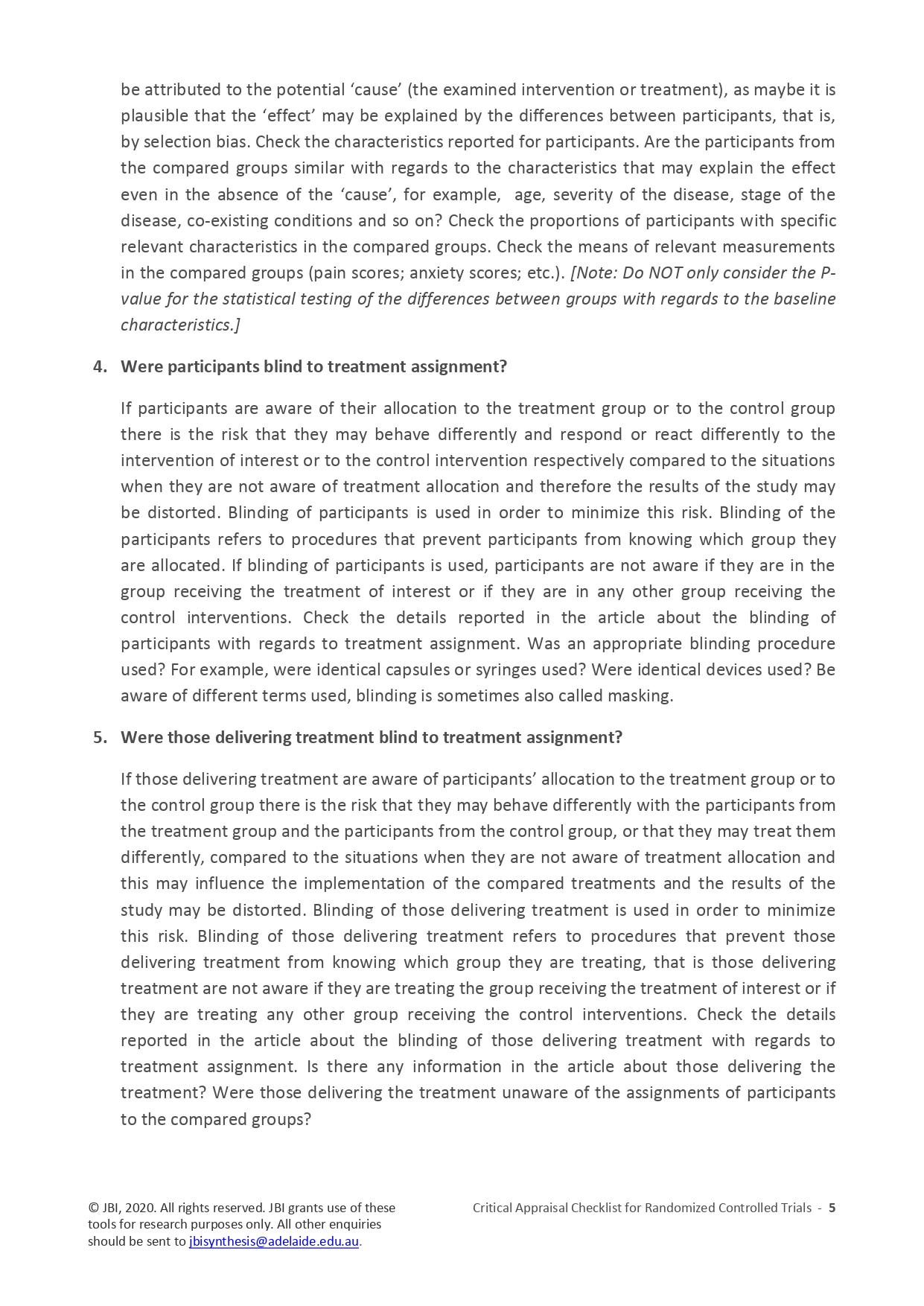

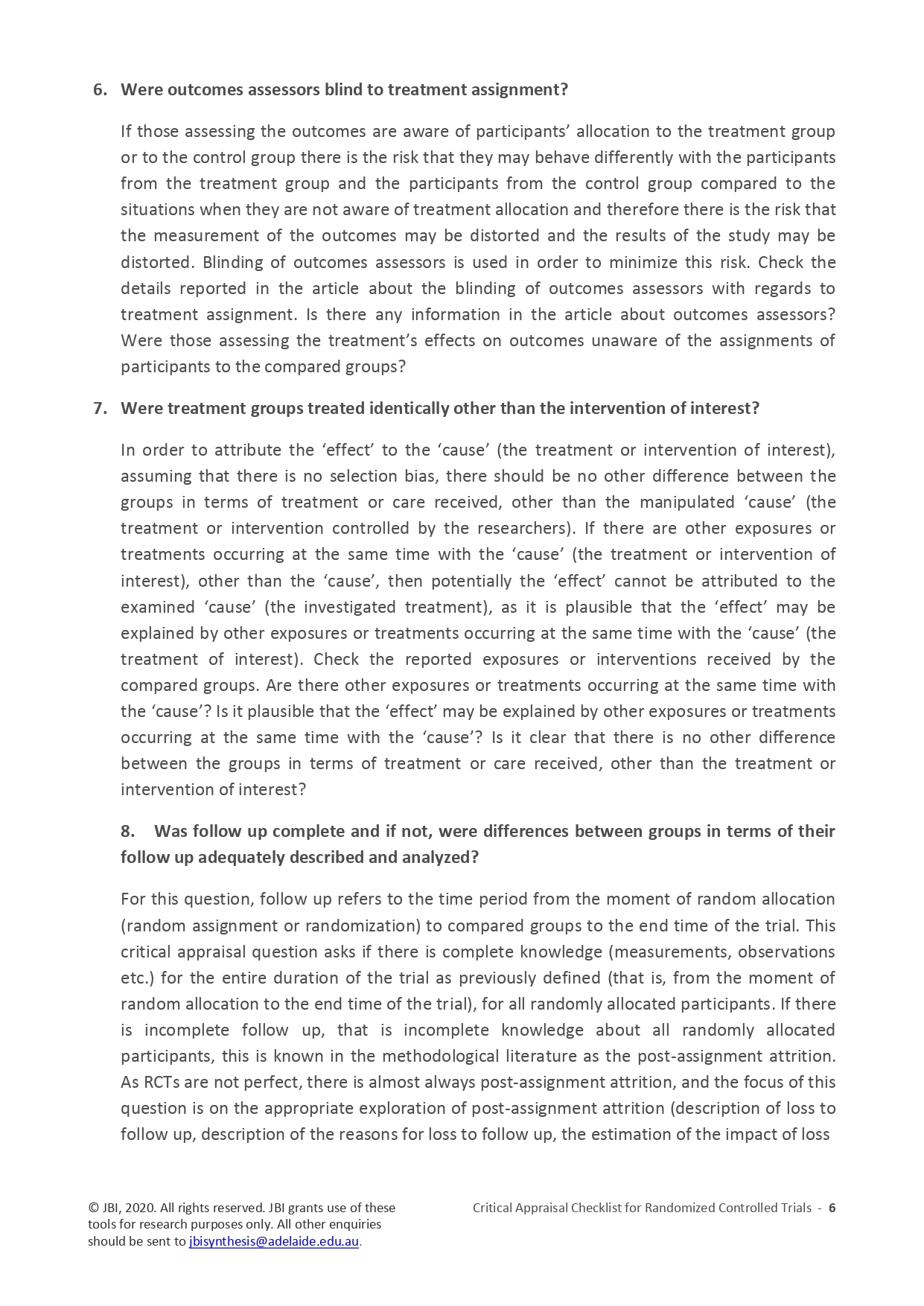


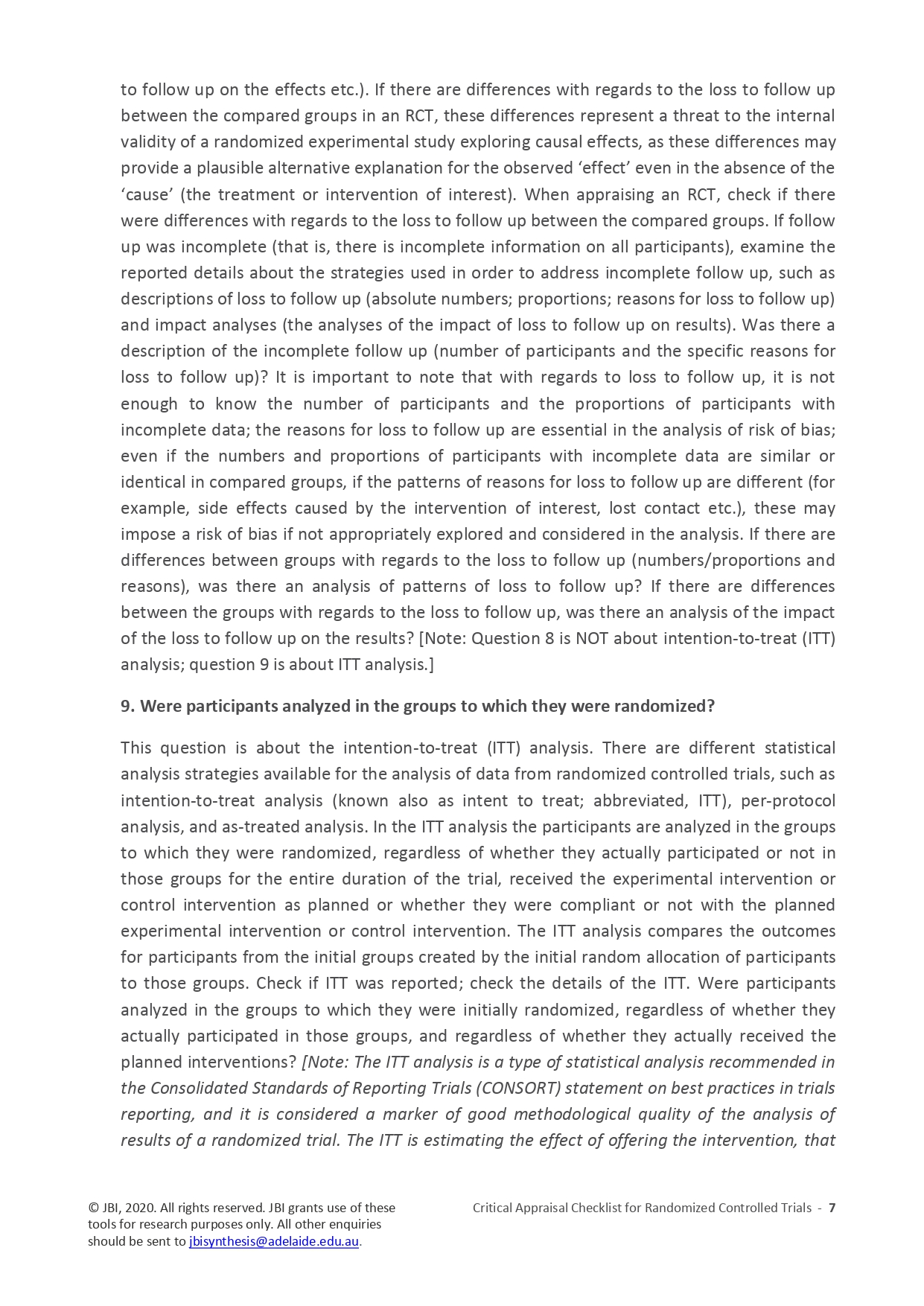

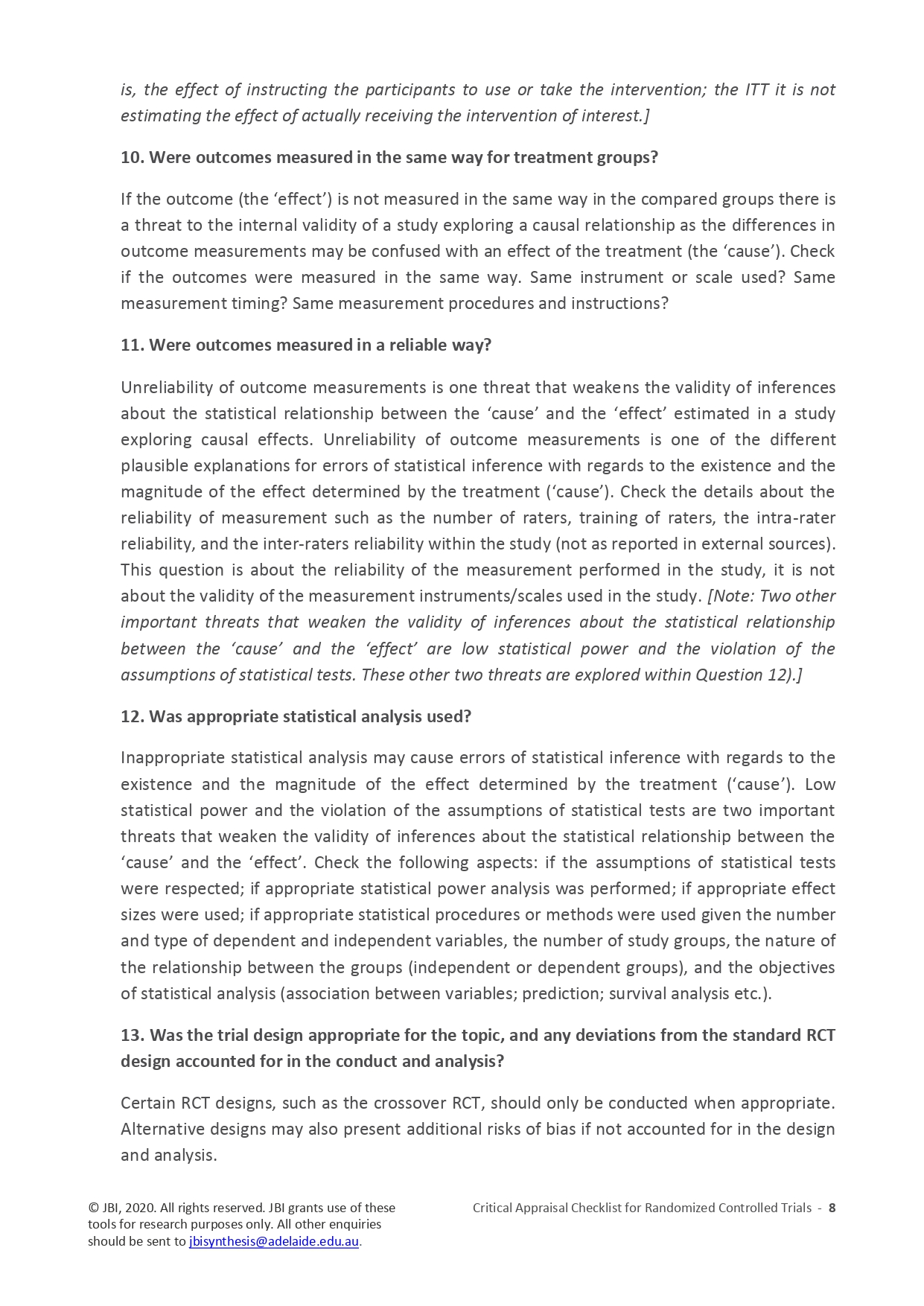

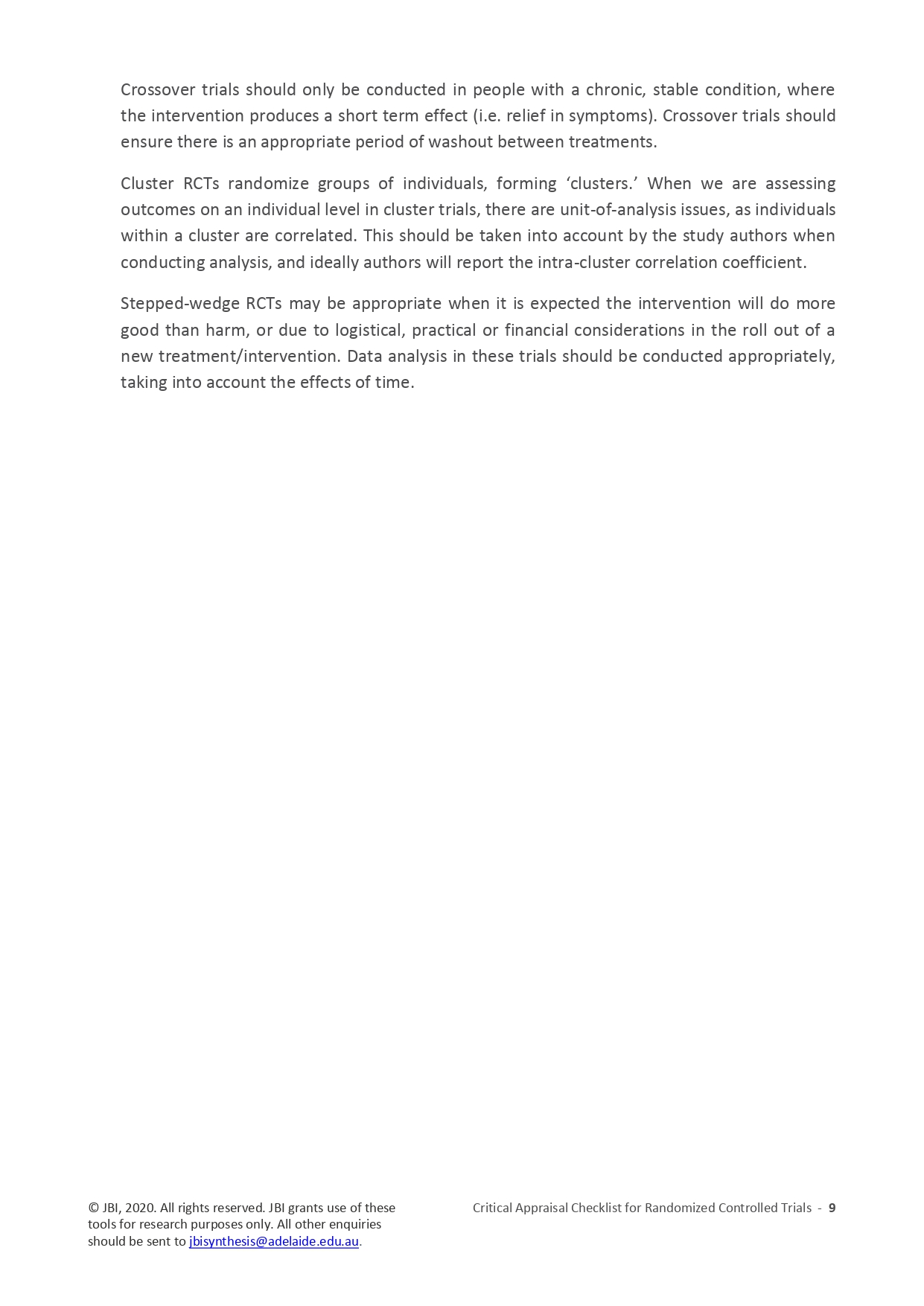

Supplement: S5 Appendix — (DOCX) [file pone.0289379.s005.docx]
