# Supplementary figures and images for: Incident sarcopenia in hospitalized older people: A systematic review
Source: PLoS One. 2023 Aug 2;18(8):e0289379. doi: 10.1371/journal.pone.0289379 (PMC10395895; doi:10.1371/journal.pone.0289379)

**Appendix 6. AMSTAR2 Checklist**


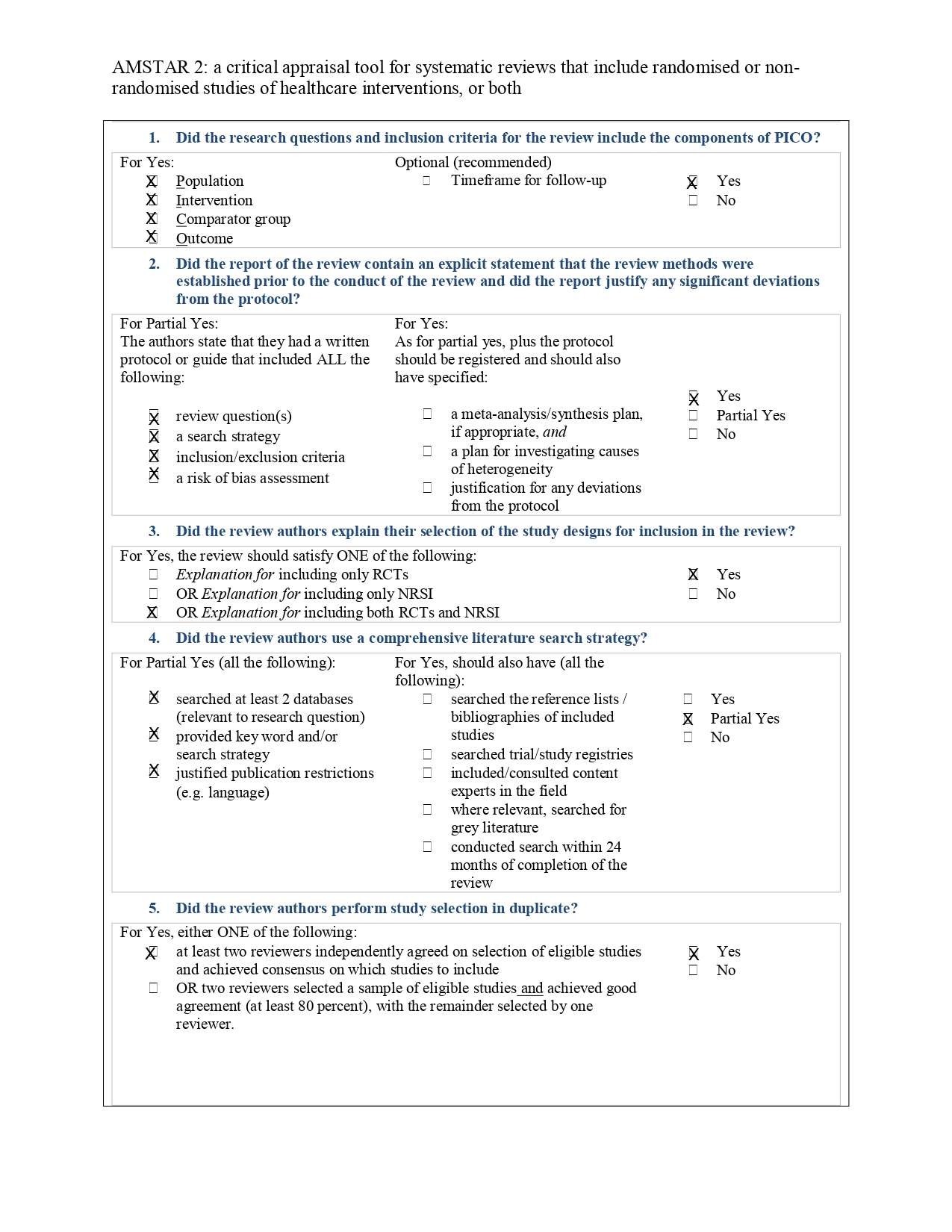


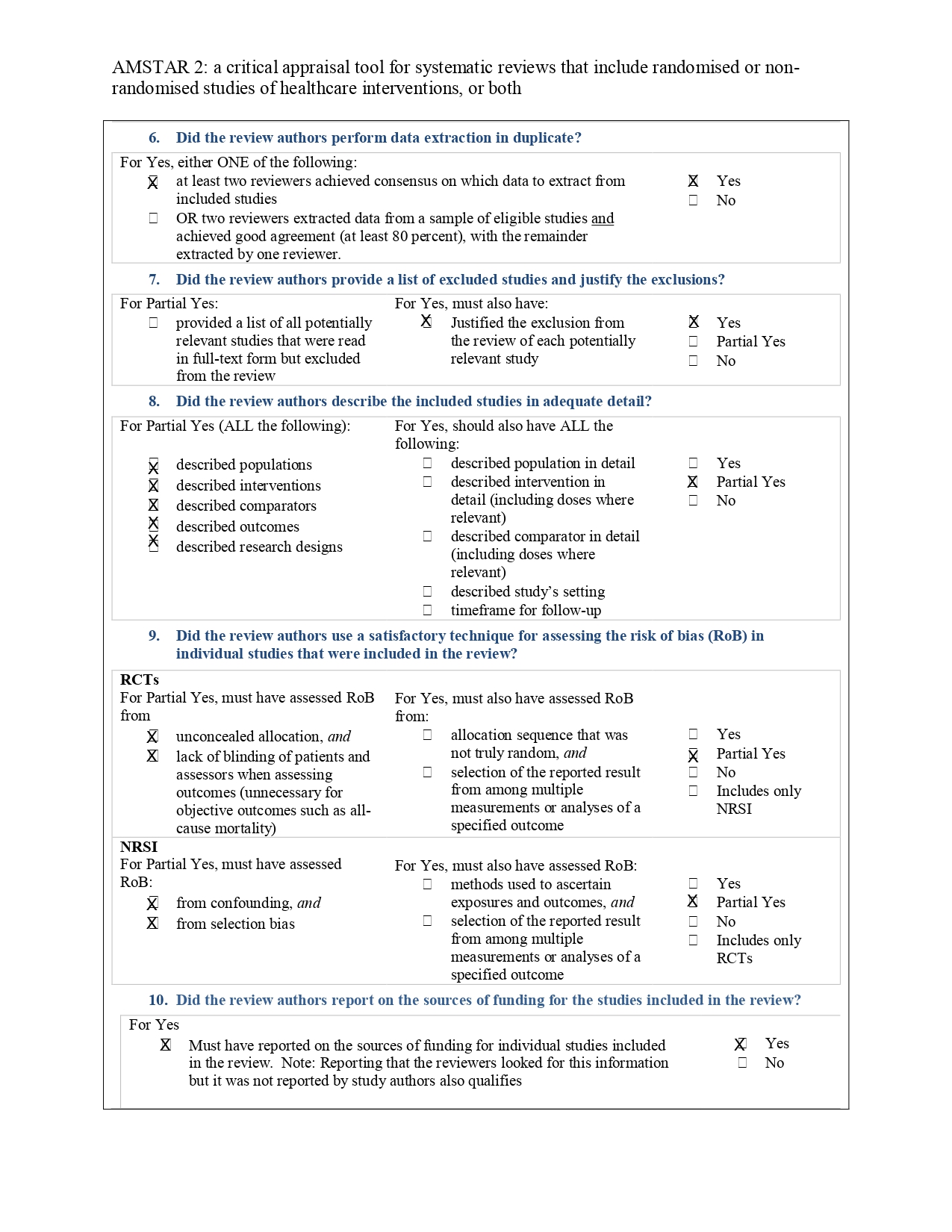


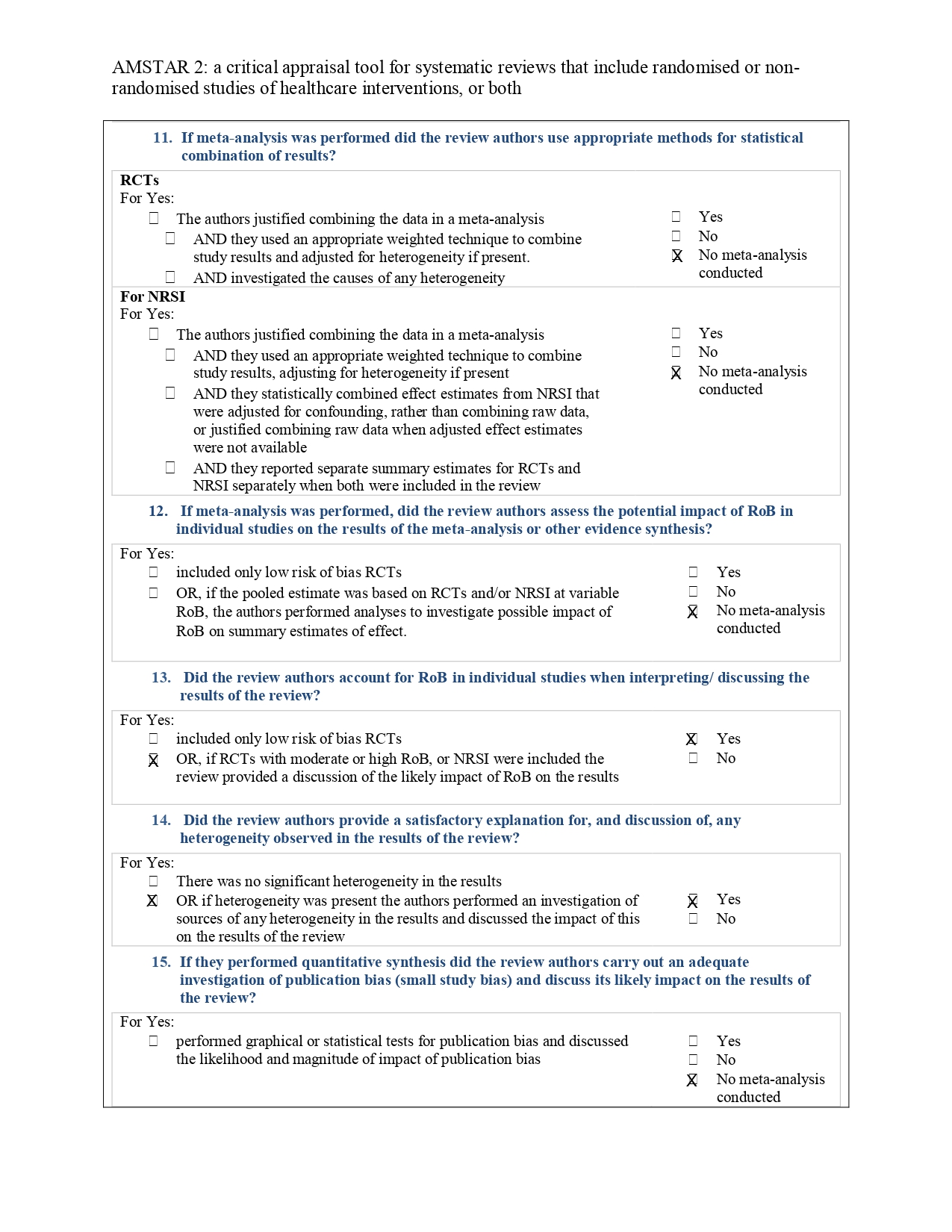


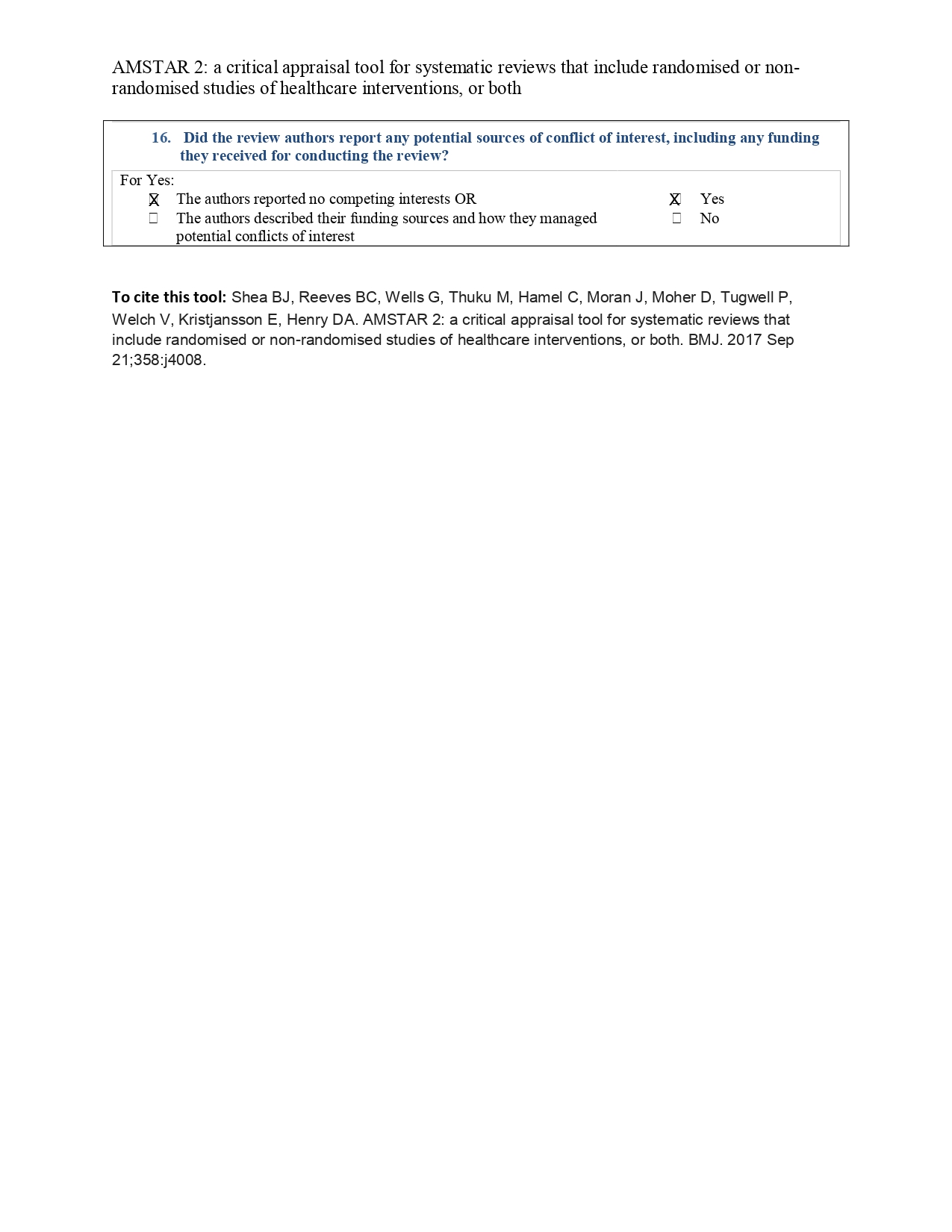

Supplement: S6 Appendix — (DOCX) [file pone.0289379.s006.docx]
